# Design Rules of the Mixing Phase and Impacts on Device Performance in High-Efficiency Organic Photovoltaics

**DOI:** 10.34133/2022/9817267

**Published:** 2022-07-26

**Authors:** Jingnan Song, Ming Zhang, Tianyu Hao, Jun Yan, Lei Zhu, Guanqing Zhou, Rui Zeng, Wenkai Zhong, Jinqiu Xu, Zichun Zhou, Xiaonan Xue, Chun-Chao Chen, Weihua Tang, Haiming Zhu, Zaifei Ma, Zheng Tang, Yongming Zhang, Feng Liu

**Affiliations:** ^1^School of Chemistry and Chemical Engineering, Frontiers Science Center for Transformative Molecules, Center of Hydrogen Science, Shanghai Key Lab of Electrical Insulation & Thermal Aging, Shanghai Jiao Tong University, Shanghai 200240, China; ^2^Department of Physics, Imperial College London, London SW7 2AZ, UK; ^3^School of Materials Science and Engineering, Shanghai Jiao Tong University, Shanghai 200240, China; ^4^Institute of Flexible Electronics (IFE, Future Technologies), Xiamen University, Xiamen 361005, China; ^5^Department of Chemistry, Zhejiang University, Hangzhou 310027, China; ^6^Center for Advanced Low-Dimension Materials, State Key Laboratory for Modification of Chemical Fibers and Polymer Materials, College of Materials Science and Engineering, Donghua University, Shanghai 201620, China; ^7^State Key Laboratory of Fluorinated Functional Membrane Materials and Dongyue Future Hydrogen Energy Materials Company, Zibo City, Shandong Province 256401, China

## Abstract

In nonfullerene acceptor- (NFA-) based solar cells, the exciton splitting takes place at both domain interface and donor/acceptor mixture, which brings in the state of mixing phase into focus. The energetics and morphology are key parameters dictating the charge generation, diffusion, and recombination. It is revealed that tailoringthe electronic properties of the mixing region by doping with larger-bandgap components could reduce the density of state but elevate the filling state level, leading to improved open-circuit voltage (*V*_OC_) and reduced recombination. The monomolecular and bimolecular recombinations are shown to be intercorrelated, which show a Gaussian-like relationship with *V*_OC_ and linear relationship with short-circuit current density (*J*_SC_) and fill factor (FF). The kinetics of hole transfer and exciton diffusion scale with *J*_SC_ similarly, indicating the carrier generation in mixing region and crystalline domain are equally important. From the morphology perspective, the crystalline order could contribute to *V*_OC_ improvement, and the fibrillar structure strongly affects the FF. These observations highlight the importance of the mixing region and its connection with crystalline domains and point out the design rules to optimize the mixing phase structure, which is an effective approach to further improve device performance.

## 1. Introduction

The characteristics of the tightly bonded exciton [1, 2], morphology-dependent carrier kinetics [3, 4], and hopping type transport [5–7] have made organic photovoltaic (OPV) very different from traditional silicon solar cells. For morphology-dependent carrier kinetics, the spontaneous phase-separation in-between donor (D) and acceptor (A) molecules introduces D/A interfaces for splitting excitons and bicontinuous networks for transporting charge carriers to corresponding electrodes [8–10]. For optimized morphology, both D and A molecules are required to form crystalline domains that dramatically improve the carrier transport of both electrons and holes [11–15]. On opposite, when D/A mixture fails to form crystalline domains, but forms an amorphous mixing region, the photogenerated carriers suffer serious recombination before reaching the electrodes [16, 17]. Thus, the study of D/A morphology finds its unique importance in OPV research.

Recently, the emerging of nonfullerene acceptors (NFAs) has greatly improved the power conversion efficiency (PCE) of OPV. Unlike fullerene derivatives, NFA usually mixes well with donor molecules to induce a large content of amorphous mixing domain in morphology, away from spinodal decomposition created nanostructure [18–20]. As a result, the mixing zone of D/A becomes the key morphological issue that needs to be further optimized to obtain high PCE in NFA-based OPVs. From morphology perspective, the photon-generated carriers have to escape from the mixed zone of D/A and reach the crystalline region before they recombine [10, 21, 22]. Thus, the appropriate size and interface energy levels of the mixed zone that suit photon-to-electron conversion process is critical in NFA-based OPVs.

The current work takes the initiative to access the morphology and electronic structure of the mixing region and summarizes the impact of optoelectronic process on device performance to point out the design route. It is seen that PM6 and Y6 components form good crystalline domains that establish a multilength-scale phase separation [23]. Here, PM6:Y6, as the model system, was used as the high-efficiency platform [24], and a small amount of ITIC-type acceptors was introduced to dope into the mixing region. A large library of ternary blends was constructed where the morphology and electronic structure were dependent on the physical properties of the secondary acceptors. We find that the ternary blends possess tunable nonradiative energy loss with good coordination with *V*_OC_, indicating that the nonradiative recombination is the main energy loss channel in the mixing phase. The electronic state of the mixing phase was characterized by density of states (DoS). It was found that the density of the electron filling states changed upon different secondary acceptors, resulting in different open-circuit voltage (*V*_OC_). The secondary acceptor doping displayed a *g*-factor of ~20% in lamellae packing direction for Y6 molecules, meaning that the structure order was reduced for Y6, yet could still act as a suitable *n*-type transport media. The close interaction of the secondary acceptor with PM6 and Y6 effectively changes the electronic properties of the mixing region, offering an effective optimization methodology in manipulating charge transfer and carrier diffusion. The optimized ternary blends afford a maximum device efficiency of ~17.5% with simultaneous improved short-circuit current density (*J*_SC_), *V*_OC_, and fill factor (FF), owing to balanced morphology and electronic properties, which is found cross-correlated with photophysical behaviors, revealing the fundamental mechanism of device operation.

## 2. Results and Discussions

**Figure 1:**
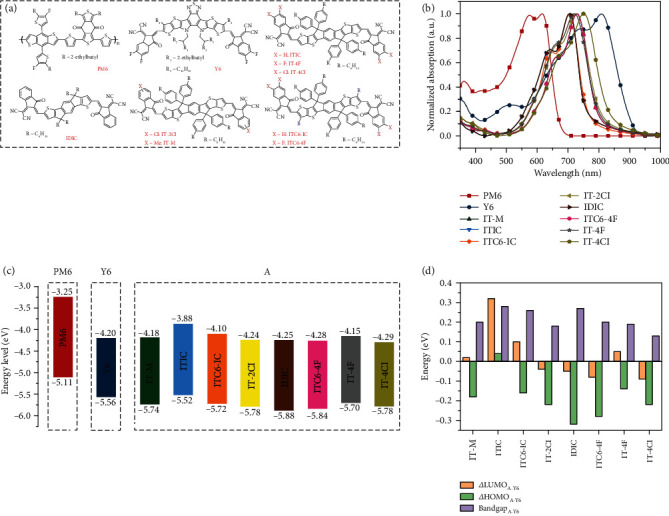
Properties of the materials. (a) The chemical structure, (b) normalized UV-vis absorption, and (c) energy level of the materials used in the measurement and device fabrication. (d) The difference of LUMO, HOMO, and bandgap between the third components and Y6.

Figure 1(a) shows the chemical structure of the materials used in the study (PM6, Y6, and ITIC-type acceptors). The corresponding UV-vis absorption spectra are shown in Figure 1(b). The energy level diagrams are shown in Figure 1(c), built by using the ultraviolet photoelectron spectra (UPS, Figure S1) to measure the highest occupied molecular orbital (HOMO) level and subtracting the optical bandgaps to obtain the lowest unoccupied molecular orbital (LUMO) level. The modification of the alkyl side chains and the end group substitution could fine-tune the frontier energy levels on both hole and electron sides, providing a good handle to manipulate the electronic structure. The energy level differences referenced to the major acceptor Y6, including *Δ*HOMO, *Δ*LUMO, and Δ*E*_*g*_, are summarized in Figure 1(d), which could be used as the key variables to assess the physical properties of the ternary blends.

**Figure 2:**
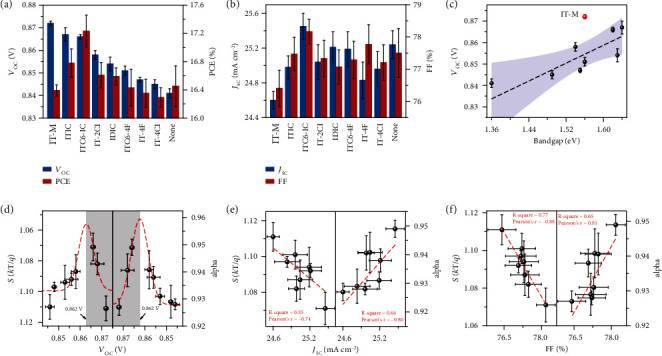
Photovoltaic performance and recombination analysis. (a) The PCE and *V*_OC_ and (b) *J*_SC_ and FF of the devices with different ITIC derivatives as the third component (40 devices average). (c) *V*_OC_ of the ternary device versus the bandgap of the third component showing positive correlation with confidence zone purple marked. The IT-M is marked in red and does not participate in the fitting due to its large deviation. (d) The relationship between the light-dependent parameters and *V*_OC_, which shows a Gauss-like distribution with the peaks both located at ~0.862 V. The relationship between the light-dependent parameters and (e) *J*_SC_ and (f) FF both show linear regression with R-square and Pearson's *r* inset.

Solar cells were fabricated to evaluate the performances of devices with different secondary acceptors using the forward device structure of ITO/PEDOT:PSS/PM6:Y6:A/PFNDI-Br/Ag. The donor to acceptor ratio was fixed at 1 : 1.2, and the ratio of Y6:A was kept at 1 : 0.2. The low concentration of the secondary acceptors led to good mixing in the amorphous region, and their crystallization could be ignored. The current density-voltage (*J-V*) curves of the optimal devices are shown in Figure S2, and the corresponding photovoltaic parameters and statistical data are summarized in Figures 2(a) and 2(b) and Table 1. The PM6:Y6 binary device showed a maximum PCE of 16.74% with *V*_OC_ of 0.843 V, *J*_SC_ of 25.36 mA cm^−2^, and an FF of 78.32%. The introduction of the secondary acceptors significantly influenced the device performances. The device *V*_OC_ showed a satisfying improvement due to the incorporation of a large-bandgap acceptor. The built-in potential of the device got increased, and the highest *V*_OC_ of 0.871 V was obtained in PM6:Y6:IT-M device. *J*_SC_ and FF vary on different material blends, which is associated with carrier transport and recombination due to the difference in morphology and electronic structure. The best system (PM6:Y6:ITC6-IC) achieved a PCE of 17.46%, with simultaneous improvements in *V*_OC_, *J*_SC_, and FF (Figure S3 and Table S1). The PCE of the device was certified as 17.03%, subject to the calibration procedures of the National Renewable Energy Laboratory (NREL), using a 0.032 cm^2^ photon mask (Figure S4). *J*_SC_ was further checked by integrated EQE (Figure S5), yielding good agreements. The *V*_OC_ dependence on the bandgap of the secondary acceptors is shown in Figure 2(c), where the solid line is the linear fitting curve with the confidence zone marked in gray. A proportional relationship is observed; thus, the secondary acceptor plays a critical role in thin film electronic structure. These results reveal a quite effective approach in *V*_OC_ manipulation by optimizing the heterojunction energetics and reducing the exciton and carrier recombination in the mixing region. The good miscibility between the acceptors (Figure S6) and the low concentration doping method shift the argument of device operation away from the parallel model, where each acceptor should form its own isolated transport phase without charge or energy transfer [25–27]. Such modification is effective to improve the diode characteristics, thus achieving increased FF simultaneously. It should be noted that the exciton and carrier kinetics in the mixed region is hard to be summarized using a simple variable. Exciton splitting and charge transfer, charge transport, and recombination are entangled with the blend morphology, which leads to the fluctuations and a broad confidence zone in the current investigation. This will be discussed in the following section in detail.

Light intensity-dependent *V*_OC_ and *J*_SC_ measurement were carried out. A slope from *V*_OC_ vs. *P*_light_ of 2 kT/q should be obtained (expressed as the ideality factor *S*) if monomolecular or trap-assisted recombination dominates. The recombination parameter *α*, defined by *J*_SC_ ~ (*P*_light_)^*α*^, is close to unity, suggesting minimal recombination, indicating effective free charge collection at short circuit condition [28, 29]. *S* and *α* values were extracted through linear fitting with the corresponding results shown in Figure S7, and the relationship between *V*_OC_ and *S*, *α* is shown in Figure 2(d). The modification of the mixing region by acceptor mixture leads to unexpected device behavior. A nearly symmetric Gaussian-type distribution for *S* and *α* is seen, and the center of the Gaussian distribution locates at ~0.862 V, where the recombinations reach a minimum. It is indicated that recombination influences *V*_OC_ in multiple pathways either in exciton splitting or carrier transport, which leads to this type of unordinary nonlinear correlation. This result is intriguing, indicating that the monomolecular and bimolecular recombinations are intercorrelated, and the best affordable *V*_OC_ for the PM6:Y6 blends is ~0.862 V due to the constraint between *V*_OC_ loss and recombination rate [30, 31]. Cases from previous reports are provided in Table S2, yet the mechanism still needs further research. However, it is concluded that the carrier transport in the mixing region is as important as that in the crystalline domains. Thus, a global morphology optimization should consider both aspects into account. *J*_SC_ and FF are inversely correlated with recombinations, which are shown in Figures 2(e) and 2(f). For *J*_SC_, higher correlation constant is seen for *α*, and thus, reducing recombination is key. For FF, both *S* and *α* correlations are strong, and the *S* correlation is slightly larger, thus reducing the shallow traps and carrier annihilation are important. These correlations summarize not only the crystalline feature in the blended thin films but also the mixing region properties, and the carrier diffusion from the mixing region can be a rate-limited process comparing to that from crystalline domains. The maximum *V*_OC_ condition is coalesced with serious monomolecular and bimolecular recombinations, and the experimental maximum PCE is obtained close to the optimized *V*_OC_ condition, indicating that *V*_OC_, *J*_SC_, and FF contain internal restraints and need to be balanced to achieve a higher PCE. It should be noted that the monomolecular recombination (*S*) and bimolecular recombination (*α*) are correlated since *dV*_OC_/*dlnG* = (*dV*_OC_/*dlnJ*_SC_)(*dlnJ*_SC_/*dlnG*). And it is physically plausible since the monomolecular recombination looks into the carrier quenching due to the existence of traps and defects that mainly takes place in the mixing region. The similar and symmetric behavior of *S* and *α* indicates that carrier diffusion in mixing region dominates the recombination in device.

**Figure 3:**
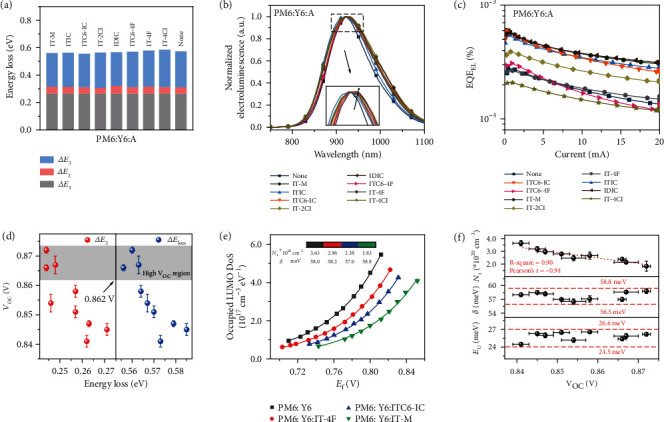
The energy loss and energetic disorder. (a) The energy loss, (b) normalized EL spectra, and (c) EQE_EL_ spectra of the ternary devices with different third components. The inset is the enlarged view of the region at ~920 nm. (d) The relationship between *V*_OC_ and different energy loss parts from radiative and nonradiative channels with high *V*_OC_ region gray marked. (e) The derived LUMO DoS from the capacitance spectra of optimal devices, exhibiting an exponential shape, where *N*_t_ is the total density per unit volume, and *δ* the energetic disorder parameter. (f) The relationship between *N*_t_, *δ*, *E*_U_, and *V*_OC_, where *E*_U_ is the Urbach energy extracted from the low energy tail of the s-EQE spectra.

It is well recognized that the electronic structure determines *V*_OC_ of OPV devices. In the current case, we doped large-bandgap materials into a narrow-bandgap mixture, and the *V*_OC_ change is thus correlated with the heterojunction interfacial energy levels. The highly sensitive EQE (s-EQE), electroluminescence (EL), and electroluminescence quantum efficiency (EQE_EL) measurements were performed to assess the energy loss [32, 33]. The total energy loss (*E*_loss_) could be calculated following *E*_loss_ = *E*_gap_ − *eV*_OC_, where *E*_gap_ is the electronic bandgap calculated on the basis of the intersections between the normalized EQE and EL spectra as shown in Figure S8. The detailed *E*_loss_ components were quantitatively analyzed, which could be classified into three different constituents (*E*_loss_ = ∆*E*_1_ + ∆*E*_2_+∆*E*_3_) as shown in Figure 3(a). ∆*E*_1_ is defined as the difference between *E*_gap_ and the Shockley-Queisser (SQ) limit output voltage (*V*_OC_^SQ^), which is caused by the radiative recombination loss above the bandgap, while ∆*E*_2_ is the radiative recombination loss below the bandgap. ∆*E*_3_ originates from the nonradiative recombination loss, which can be directly calculated from the EQE-EL spectra as defined by ∆*E*_3_ = −*kT*ln(EQE_EL_), associating with tail electronic states and the nonradiative recombination. These results are summarized in Table S3, and the EL spectra and EQE_EL_ are shown in Figures 3(b) and 3(c). For PM6:Y6 binary device, a single emission peak is seen at ~920 nm, which is similar to that of the Y6 single-component device (920 nm). The EL emission peak for ternary blends slightly changes to ~930 nm. Meanwhile, the EL quantum efficiencies are close to or higher than PM6:Y6 device. Thus, the new mixture adding a large-bandgap dopant slightly reduces the emission bandgap as well as the nonradiative recombination. We ascribe this result to the CT and singlet hybridization and reduced density of states (DoS), which in combination gives rise to lower recombination [30, 34]. The detailed *V*_OC_ and subsector *E*_loss_ correlations are shown in Figure 3(d). ∆*E*_loss_ and ∆*E*_3_ are in the same trend, indicating that nonradiative recombination is the main energy loss channel in the mixing region. The high *V*_OC_ region is highlighted with a 0.862 V bar, above which the recombination retards *J*_SC_ and FF, although the energy loss is small. The result indicates that *E*_loss_ optimization needs to be in combination with carrier generation and transport process optimization to achieve the best balance. The radiative energy loss (∆*E*_1_ + ∆*E*_2_) does not show good correlation with *V*_OC_ as shown in Figure S9, however with a roughly upward *V*_OC_ trend when radiative energy loss increases. It should be noted that an ideal OPV device is also a good LED equipment and this kind of radiative energy loss is inevitable. Manipulating the electronic properties in the mixing region to seek inhibitory route for nonradiative energy loss is an effective avenue for *V*_OC_ improvement [35]. The bimolecular and trap-assisted recombinations from the light-dependent measurements are associated with nonradiative (∆*E*_3_) losses, and each recombination kinetic factor is intrinsically correlated with electronic structure. Thus, *E*_loss_ optimization is in essence with the electronic structure optimization. We use impedance spectroscopy (IS) to access the electron density of state (DoS) of the devices, which we think is currently the best way to probe the thin film electronic properties. The DoS (*g*_*n*_) is obtained by *C*_*μ*_^*n*^ = *q*^2^∙*g*_*n*_(*E*_*Fn*_), where *C*_*μ*_^*n*^ equals to *C*/(*L*∙*S*) (*C* is the capacitance from the IS measurement, as shown in Figure S10, and *L* and *S* are the thickness and area of the device) and *q* is the elemental charge. An exponential shape is seen in Figure 3(e), typically reflecting the occupied electron states in LUMO DoS. The curves are fitted by *g*_*n*_(*E*_*Fn*_) = (*N*_*t*_/*δ*)exp(−(*E*_*g*_ − *E*_*Fn*_)/*δ*), to extract the total density per unit volume *N*_*t*_ and the width of the DoS *δ* that describes energetic disorder [36, 37]. The fitting results are summarized in Table S4. As shown in Figure 3(f), the small amount of secondary acceptor does not introduce energetic disorder, as the similar *δ* values are seen with the variation of ~2 meV. The *N*_*t*_ values show a large difference and a good negative correlation with *V*_OC_, indicating that the LUMO DoS changes with the mixing zone. Thus, as seen in Figure 3(f), in the case of PM6:Y6:IT-M device, the electronic properties of the third component and the suppressed nonradiative combination induce the low *N*_*t*_ value, which enables an easy occupation of the electrons to the higher energy level to obtain a higher effective *V*_OC_. The Urbach energy (*E*_U_) of the device was further investigated as shown in Figure S11, yielding similar values in binary and ternary devices, suggesting that the complex mixing only changes the electron states filling, but not disturb the energetic disorder.

**Figure 4:**
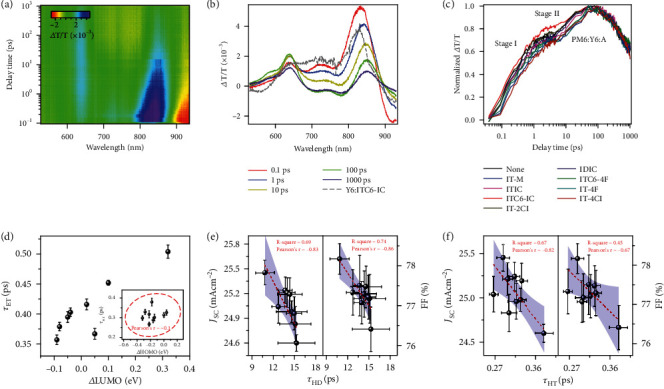
The hole and electron transfer kinetics. (a) Color plot of fs transient absorption spectra of blended film at indicated delay times under 750 nm excitation with a fluence below 10 *μ*J/cm^2^. (b) Representative fs TA spectra of blended films at indicated delay times with signal of Y6:ITC6-IC as the gray dotted lines shown. (c) The hole transfer kinetics of the binary and ternary blends, where in stage I the excitons dissociate directly and in stage II the exciton diffusion in the crystalline domain occurs. (d) The relationship between *Δ*LUMO and electron transfer time. The relationship between *Δ*HOMO and hole transfer time is shown inset with confidence ellipse plot, in which a more narrowed ellipse shape indicates a stronger interdependence. The effect of (e) hole diffusion and (f) transfer time on the device performance.

We then look into the exciton and carrier kinetics that are the key parameters coupled with the morphology and electronic properties of the blended thin films. Femto-second transient absorption spectroscopy (TAS) was used to probe the photo-induced hole and electron transfer kinetics. The hole-transfer process was accessed by selectively exciting the acceptors using a pump laser of 750 nm. Representative 2D TAS images and time-delayed profiles are shown in Figure S12. Taking PM6:Y6:ITC6-IC for example (Figures 4(a) and 4(b)), clear bleaching peaks appear between 600 and 900 nm, which is similar to the TAS signal of Y6:ITC6-IC (gray dotted lines in Figure 4(b) and Figure S13), indicating these signals are from the acceptors. With the decay of these peaks, a new peak at ~585 nm gradually emerges, in good accordance with the TAS spectra of the neat PM6 film, as shown in Figure S14a. Thus, hole-transfer from the acceptors to PM6 takes place. The rising kinetics of PM6 bleach signal was extracted, which clearly showed two stages. For the exciton generated in the mixing phase, it could rapidly dissociate due to the existence of abundant D/A molecular interface, which corresponds to stage I. For the exciton generated in the crystalline phase, it should first diffuse towards the interface of the crystalline domain and then dissociate to produce free carriers. Thus, it should sequentially experience stage II and stage I. By fitting the curves with biexponential function, the time constant for each stage can be obtained, which could identify the exciton kinetics occurred in the crystalline (*τ*_HD_) and mixing phase (*τ*_HT_) [38]. The electron transfer from donor to acceptor was investigated using a 520 nm laser excitation (Figure S15 and Figure S16), and the electron transfer time (*τ*_ET_) was recorded with the results summarized in Table S5. The correlation between charge-transfer time, energy levels referencing to Y6 (*Δ*LUMO/*Δ*HOMO, the energy level difference of the secondary acceptor comparing to that in Y6, which is summarized in Figure 1(d)), and device characteristics are shown in Figures 4(d)–4(f). It is seen that the electron transfer time (*τ*_ET_) is correlated to *Δ*LUMO (Figure 4(d)), indicating lower energy dip in acceptors plays an important role in directing the kinetics of electron transfer. The correlation between the hole-transfer time and *Δ*HOMO is poor (inset in Figure 4(d)), which is due to the fact that the 750 nm excitation can activate both acceptors and result in simultaneous hole-transfer to PM6. Shown in Figure 4(e) is the relationship between exciton diffusion time (*τ*_HD_) and *J*_SC_, FF, which is used to quantify the exciton kinetics at the crystalline domain interface. A reduced diffusion time could simultaneously improve *J*_SC_ and FF. The hole-transfer time (*τ*_HT_) and its influences on *J*_SC_ and FF are summarized in Figure 4(f), which looks into the quick exciton splitting in the mixing region. It is seen that both *J*_SC_ and FF inversely correlated with *τ*_HT_, and the *J*_SC_ correlation constant is higher. *τ*_HT_ and *τ*_HD_ behaviors reveal that exciton splitting at both crystalline domain interface and mixing region contributes to *J*_SC_ of equal importance. The good correlation of exciton diffusion and FF can be the result that better crystalline order supports good transport of both excitons and charge carriers. In this scenario, a good BHJ morphology should consider both mixing region property and thin film crystallinity, and thus, both carrier generation and transport can be optimized simultaneously.

**Figure 5:**
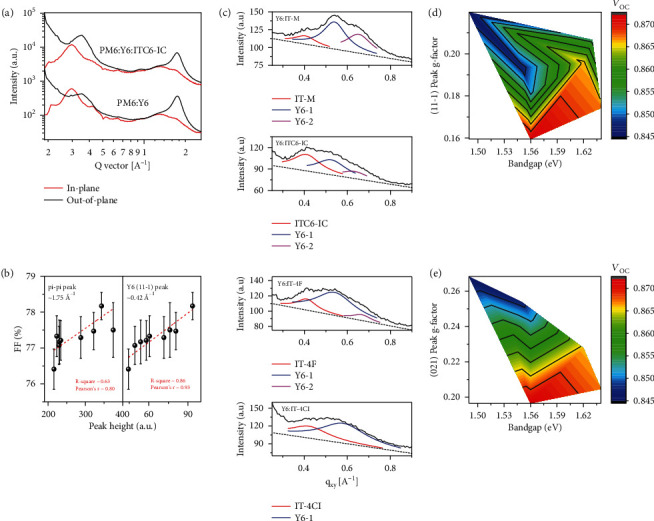
The morphology characterization. (a) The in-plane (IP) and out-of-plane (OOP) line cuts of the GIWAXS 2D patterns for ternary blends. (b) The relationship between the height of pi-pi stacking peak and Y6 (11-1) peak of the ternary films and the FF of corresponding devices. (c) The low *q* region of the 75° line cut profiles of the acceptor-only films with Lorentzian fitting results (red line for the third component, blue line for Y6 (021) peak, and purple line for Y6 (111) peak). (d) The 2D relevance between *V*_OC_ and the bandgap of ITIC derivatives, *g*-factor of Y6 (11-1) peak in IP direction of acceptor-only films. (e) The 2D relevance between *V*_OC_ and the bandgap of ITIC derivatives, *g*-factor of Y6 (021) peak in 75° line cuts of acceptor-only films. The color bar is used to reflect the value of *V*_OC_ in the 2D map, and the change from blue to red represents the *V*_OC_ improvement.

We then carried out grazing incidence wide-angle X-ray scattering (GIWAXS) to study the amorphous and crystalline features of the neat thin films, with the corresponding results shown in Figures S17 and S18. The PM6 donor assumed a dominant face-on orientation, with a broad (100) reflection in the in-plane (IP) direction at 0.28 Å^−1^ and a pi-pi stacking peak in the out-of-plane (OOP) direction at 1.69 Å^−1^. Y6 films showed good crystalline features with (110), (020), and (11-1) diffraction spots clearly seen in the 2D diffraction patterns. The ITIC-type acceptors show varied crystalline feature. For example, the crystallization of ITC6-IC, ITC6-4F, and IT-4F is very weak, while the others show characteristic lamellae peak in the IP direction and pi-pi stacking peak in the OOP direction. For the PM6:Y6 binary blend, the (020) and (11-1) peaks are suppressed, and the lamellar (~0.3 Å^−1^) and pi-pi stacking (~1.7 Å^−1^) peaks become dominant. In the ternary blends, similar diffraction patterns are seen with no crystalline signal from the third component (Figure S19), confirming that they are dissolved in mixing region. Representative IP and OOP line cuts of the PM6:Y6 and PM6:Y6:ITC6-IC blends are shown in Figure 5(a). It is seen that the Y6 (020) and (11-1) diffraction peak intensity is reduced in the ternary blends, thus reducing the stacking along the lamellae and long axis of Y6 crystallites. The correlations between FF and pi-pi stacking peak, lamellae peak, and Y6 (11-1) peak are shown in Figure 5(b) with fitting process shown in Figures S20 and S21 and peak parameters in Table S6. It is obvious that improving thin film crystallinity is beneficial for FF since the carrier transport can be improved. As shown in Figure S22, the exciton diffusion time (*τ*_HD_) shows a better correlation with Y6 (11-1) peak area than the CCL calculated from the FWHM, indicating that optimizing the thin film crystallinity (suppressing the amorphous content) to increase the carrier transport are more favorable for *J*_SC_ and FF improvement. This result supports the discussion in TAS analysis. It should be noted that in most ternary blends the relative crystallinity of Y6 slightly reduces. However, the benefit of this approach is the modification of the electronic properties of the mixing region, either in interfacial energy levels or carrier diffusion. And in the case of PM6:Y6:ITC6-IC blend where both thin film crystallinity and mixing region property are optimized, the device performance reached the maximum. We then investigated the mixing morphology of the acceptor blends (Y6 : A = 1 : 0.2, corresponding fitting process are shown in Figure S23). The Y6 lamella diffraction peaks in acceptor blends become less pronounced (Figure S24). However, the pi-pi stacking peak remains similar. Thus, the second acceptor interacts with Y6 via the alkyl chain interaction, and the carrier hopping pathways are not significantly disturbed. We calculated the *g*-factor for (11-1) peak that highlights the long axis of crystal fibril packing [3, 39]. The Y6 neat film shows a value of 11%, and the Y6:A films show the *g*-factors ranging from 15% to 20% (Table S7). Thus, the Y6 polymer-like packing is disturbed upon introducing the second acceptors. We then conducted a 75° line cut to analyze the ordering of the acceptor (021) diffraction, which is also the periodic structure induced by pi-pi interaction confirmed by single crystal [23], with results shown in Figure 5(c) and Figure S25, where Y6-1 and Y6-2 represent (021) and (111) peaks, respectively. It is seen that the secondary acceptors could form a broad and weak diffraction signal with *g*-factor of ~30%, which is amorphous, and the crystallization of Y6 is also affected. In the Y6:IT-M blend, a well-defined peak is still seen, while in the Y6:IT-4Cl blend, it becomes quite broad. The *g*-factor for the Y6-1 peak ranges from 19% to 27%, which is quite poor ordering value. Such phenomenon indicates that the boundary between the crystalline phase and the mixing phase is vague. The diffraction signal in GIWAXS can be generated from local structures with less strict ordering, considering the large size of the conjugated molecules. How to understand this kind of paracrystalline state or the “distorted ordering” is worthy of further research.

We then looked into the correlation of structure order with device performances. The bandgap, *g*-factor of (11-1) and (021) peaks, and *V*_OC_ correlation are shown in Figures 5(d) and 5(e). As shown in Figure 5(d), the lowest *V*_OC_ locates at the upper left corner, indicating the long axis ordering of Y6 and the bandgap of the third component interactively determine *V*_OC_ of the device. This effect can be understood that Y6 has the lowest bandgap, and the increased crystallization would reduce its concentrating in mixing zone, and thus, the higher bandgap secondary acceptor could thus play a more important role. It is interesting to see that the highest *V*_OC_ locates at the middle bottom; thus, choosing the suitable bandgap secondary acceptor while maintaining good local ordering is important in enhancing *V*_OC_. As shown in Figure 5(e), *V*_OC_ processes a better correlation with (021) peak, which is extracted cleanly without the coupling with ITIC derivatives. The highest *V*_OC_ point appears in the bottom, where the local ordering dictates. We ascribe such effect to the reduced recombination loss in more ordered blends. The amplitude of Y6 (021) peak is also extracted, which shows a good relationship with *J*_SC_ (*R*^2^ = 0.81, Pearson's *r* = −0.9) and FF as shown in Figure S26. In PM6:Y6:A blends, the secondary acceptor performs good contact with Y6 molecules in the amorphous matrix, affording new electronic states. Such change significantly changes the photophysical and electrical properties of the blended thin films. The ordering of Y6 molecules though disturbed by the secondary acceptors can still form ideal electron transport pathways, giving rise to a high FF. These observations reveal the importance of the mixing region feature on the overall morphology and photophysical process. Its structure and electronic property are key in dictating the carrier generation, transport, and recombination.

## 3. Conclusion

To conclude, we have revealed the mystery of morphology and electronic property of the mixing region and its impact on OPV devices. Multicomponent correlations regarding the recombination, transport, charge transfer, and morphology have been built to understand the strong connection between structure factor, electronic property, exciton/carrier kinetics, and device performances. The mixing domain is the ambipolar matrix that hosts donor/acceptor crystalline phase. Its interface energy, size, and molecular interaction determine the charge transfer kinetics and carrier diffusion, which strongly influence the device *V*_OC_, *J*_SC_, and FF, as shown in experimental results. In the current stage, to seek for further improvement, the features of the mixing phase need to be emphasized, which is the key region that determines energy loss and carrier recombination, as seen from the charge transfer kinetics and recombination study. The materials' interaction could be used to adjust the thin film crystallization behavior, which dictates the carrier transport in the blended thin film after the generated carriers diffuse out of the mixing region. Thus, introducing extra component with favorable energy level and suitable intermolecular interaction can be a viable approach in optimizing the morphology and electronic structure of the blended thin film, which is demonstrated here by doping with the larger-bandgap acceptors.

## Figures and Tables

**Table 1:** Photovoltaic parameters of BHJ solar cells under illumination of AM 1.5 G, 100 mW/cm^2^. The average parameters were calculated from 40 devices, with the area of 0.032 cm^2^. Values outside the parentheses denote the best optimal results.

PM6:Y6:A (1 : 1 : 0.2)	*V*_OC_ (V)	*J*_SC_ (mA·cm^−2^)	FF (%)	PCE (%)	*J*_SC_^EQE^ (mA·cm^−2^)
None	0.843 (0.841 ± 0.002)	25.36 (25.24 ± 0.16)	78.32 (77.50 ± 0.76)	16.74 (16.45 ± 0.29)	25.18
IT-M	0.871 (0.872 ± 0.001)	24.67 (24.6 ± 0.10)	76.64 (76.41 ± 0.56)	16.47 (16.39 ± 0.08)	24.35
ITIC	0.868 (0.867 ± 0.003)	25.00 (24.98 ± 0.13)	78.26 (77.47 ± 0.52)	16.98 (16.78 ± 0.20)	24.78
ITC6-IC	0.866 (0.866 ± 0.001)	25.49 (24.45 ± 0.15)	79.05 (78.17 ± 0.38)	17.46 (17.23 ± 0.23)	25.30
IT-2Cl	0.858 (0.858 ± 0.002)	25.10 (25.04 ± 0.20)	77.99 (77.33 ± 0.57)	16.79 (16.61 ± 0.18)	24.86
IDIC	0.856 (0.854 ± 0.003)	25.25 (25.21 ± 0.18)	77.32 (77.07 ± 0.53)	16.71 (16.59 ± 0.12)	25.11
ITC6-4F	0.853 (0.851 ± 0.002)	25.15 (25.19 ± 0.20)	77.8 (77.29 ± 0.58)	16.69 (16.43 ± 0.26)	25.05
IT-4F	0.848 (0.847 ± 0.001)	24.94 (24.83 ± 0.21)	78.20 (77.77 ± 0.61)	16.55 16.35 ± 0.20)	24.46
IT-4Cl	0.846 (0.845 ± 0.002)	24.98 (24.96 ± 0.20)	77.76 (77.21 ± 0.55)	16.43 (16.29 ± 0.14)	24.59

## Data Availability

All data are available in the main text and supplementary materials. Other relevant data are available from corresponding authors upon reasonable request.

## Supplementary Materials

Supplementary Materials Table S1: photovoltaic parameters of PM6:Y6:ITC6-IC devices with different mixing ratio of acceptors. Table S2: the *V*_OC_ values after mixing phase manipulation from some previous reports. Table S3: summary of the energy loss details for the binary and ternary devices. Table S4: summary of the fitting results of IS measurements. Table S5: summary of hole transfer time and electron transfer time derived from fitting the corresponding TA kinetics. Table S6: summary of the fitting results of BHJ devices. Table S7: summary of the fitting results of acceptor-only devices. Figure S1: UPS spectra of neat films. Figure S2: current density-voltage (*J*-*V*) curves of PM6:Y6-based binary and ternary devices. Figure S3: photovoltaic parameters of PM6:Y6:ITC6-IC devices. Figure S4: NREL certification report of PM6:Y6:ITC6-IC-based device. Figure S5: external quantum efficiency (EQE) spectra of PM6:Y6-based binary and ternary devices. Figure S6: the miscibility properties of PM6, Y6, and ITIC. Figure S7: light intensity-dependent *V*_OC_ and *J*_SC_ for PM6:Y6-based binary and ternary devices. Figure S8: electroluminescence spectra and the external quantum efficiency used to analyze the energy loss. Figure S9: the relationship between *V*_OC_ and radiative energy loss. Figure S10: the impedance spectra of the devices with different irradiation under open-circuit condition. Figure S11: the Urbach energy fitting of the energy onset of the s-EQE spectra. Figure S12: transient absorption (TA) for hole transfer kinetics. Figure S13: transient absorption (TA) for Y6:ITC6-IC film. Figure S14: transient absorption (TA) for neat films. Figure S15: transient absorption (TA) with excitation wavelength of 550 nm. Figure S16: transient absorption (TA) for electron transfer kinetics. Figure S17: 2D GIXD images and corresponding 1D GIXD profiles of neat PM6 and Y6 films. Figure S18: 2D GIXD images and corresponding 1D GIXD profiles of ITIC derivatives meat films. Figure S19: 2D GIXD images and corresponding 1D GIXD profiles of PM6:Y6 binary and PM6:Y6:A ternary blend films. Figure S20: the multiple Lorentzian fitting of the line cuts along the IP direction for the BHJ thin films. Figure S21: the multiple Lorentzian fitting of the line cuts along the OOP direction for the BHJ thin films. Figure S22: the 2D relevance between exciton diffusion time and Y6 (11-1) peak CCL and area in IP line cuts of BHJ devices. Figure S23: the multiple Lorentzian fitting of the line cuts along the IP direction for the acceptor-only thin films. Figure S24: 2D GIXD images and corresponding 1D GIXD profiles of Y6:A thin films. Figure S25: the low *q* region of the 75° line cut profiles with Lorentzian fitting results. Figure S26: the relationship between the intensity of Y6 (021) peak in the 75° direction and *J*_SC_, FF.
