# Supplementary material for: Design Rules of the Mixing Phase and Impacts on Device Performance in High-Efficiency Organic Photovoltaics
Source: Research (Wash D C). 2022 Jul 26;2022:9817267. doi: 10.34133/2022/9817267 (PMC9362714; doi:10.34133/2022/9817267)
Supplement: Supplementary Materials — Table S1: photovoltaic parameters of PM6:Y6:ITC6-IC devices with different mixing ratio of acceptors. Table S2: the VOC values after mixing phase manipulation from some previous reports. Table S3: summary of the energy loss details for the binary and ternary devices. Table S4: summary of the fitting results of IS measurements. Table S5: summary of hole transfer time and electron transfer time derived from fitting the corresponding TA kinetics. Table S6: summary of the fitting results of BHJ devices. Table S7: summary of the fitting results of acceptor-only devices. Figure S1: UPS spectra of neat films. Figure S2: current density-voltage (J-V) curves of PM6:Y6-based binary and ternary devices. Figure S3: photovoltaic parameters of PM6:Y6:ITC6-IC devices. Figure S4: NREL certification report of PM6:Y6:ITC6-IC-based device. Figure S5: external quantum efficiency (EQE) spectra of PM6:Y6-based binary and ternary devices. Figure S6: the miscibility properties of PM6, Y6, and ITIC. Figure S7: light intensity-dependent VOC and JSC for PM6:Y6-based binary and ternary devices. Figure S8: electroluminescence spectra and the external quantum efficiency used to analyze the energy loss. Figure S9: the relationship between VOC and radiative energy loss. Figure S10: the impedance spectra of the devices with different irradiation under open-circuit condition. Figure S11: the Urbach energy fitting of the energy onset of the s-EQE spectra. Figure S12: transient absorption (TA) for hole transfer kinetics. Figure S13: transient absorption (TA) for Y6:ITC6-IC film. Figure S14: transient absorption (TA) for neat films. Figure S15: transient absorption (TA) with excitation wavelength of 550 nm. Figure S16: transient absorption (TA) for electron transfer kinetics. Figure S17: 2D GIXD images and corresponding 1D GIXD profiles of neat PM6 and Y6 films. Figure S18: 2D GIXD images and corresponding 1D GIXD profiles of ITIC derivatives meat films. Figure S19: 2D GIXD images and corresponding 1D [file 9817267.f1.docx]

Design rules of the mixing phase and impacts on device performance in high-efficiency organic photovoltaics

Jingnan Song^1,8^, Ming Zhang^1,8^, Tianyu Hao^1,8^, Jun Yan^2^, Lei Zhu^1^, Guanqing Zhou^1^, Rui Zeng^1^, Wenkai Zhong^1^, Jinqiu Xu^1^, Zichun Zhou^1^, Xiaonan Xue^1^, Chun-Chao Chen^6^, Weihua Tang^4^, Haiming Zhu^3^, Zaifei Ma^7^, Zheng Tang^7^, Yongming Zhang^1,5^, Feng Liu^1,5*^

^1^School of Chemistry and Chemical Engineering, Frontiers Science Center for Transformative Molecules, Center of Hydrogen Science, Shanghai Key Lab of Electrical Insulation & Thermal Aging, Shanghai Jiao Tong University, Shanghai 200240, P. R. China.

^2^Department of Physics, Imperial College London, London SW7 2AZ, UK.

^3^Department of Chemistry, Zhejiang University, Hangzhou 310027, P. R. China.

^4^Institute of Flexible Electronics (IFE, Future Technologies), Xiamen University, Xiamen 361005, China.

^5^State Key Laboratory of Fluorinated Functional Membrane Materials and Dongyue Future Hydrogen Energy Materials Company, Zibo City, Shandong Province 256401, P. R. China.

^6^School of Materials Science and Engineering, Shanghai Jiao Tong University, Shanghai 200240, P. R. China.

^7^Center for Advanced Low-dimension Materials, State Key Laboratory for Modification of Chemical Fibers and Polymer Materials, College of Materials Science and Engineering, Donghua University, Shanghai 201620, P. R. China.

^8^These authors contributed equally: Jingnan Song, Ming Zhang, Tianyu Hao.

*Email: fengliu82@sjtu.edu.cn (F.L.)

**Supplementary information**

**Materials.** All reagents and chemicals were purchased from commercial sources (Aldrich or Acros) without further purification. PM6,Y6, ITIC, IT-M, IT-4F, IDIC, IT-2Cl and IT-4Cl were purchased from Solarmer Company. ITC6-IC and ITC6-4F were from Weihua Tang’s group. The silicon nitride membrane is from Clean -SiN Company.

**Device Fabrication.** Organic solar cell devices with ITO/PEDOT:PSS/Active Layer/PFNDI-Br/Ag regular structures were fabricated according to the following procedure. Patterned ITO glass substrates were sequential cleaned by ultrasonicating in acetone, detergent, deionized water and isopropyl alcohol for 15 min each and then dried under 80^o^C. The precleaned substrates were treated in an ultraviolet-ozone chamber for 15 min, then a ~40 nm thick PEDOT:PSS (Clevious P VP AI 4083 H. C. Stark, Germany) thin film was deposited onto the ITO surface by spin-coating and baked at 150 °C for 20 min. The blend solution with different mixing ratio (14 mg mL^-1^ in total) in CF (with 0.5% CN solvent additive) was stirred at 25 °C for 120 min in advance and then spin-coated on top of the PEDOT:PSS layer. The prepared films were treated with thermal annealing at 85^o^C for 6 min. After cooling to room temperature, a ~5 nm thick of PFNDI-Br (0.5 mg mL^-1^) was spin-coated on the top of active layer. Then, those samples were brought into to an evaporate chamber and a 140 nm thick silver layer was thermally evaporated on the PFNDI-Br layer at a base pressure of 1 × 10^-6^ mbar. The evaporation thickness was controlled by SQC-310C deposition controller (INFICON, Germany). Ten devices were fabricated on one substrate and the active area of each device was 0.032 cm^2^ defined by a shadow mask.

**General methods.** UV-vis absorption spectra were recorded on a Shimadzu spectrometer model UV-1800 with films on the quartz plates at room temperature. The morphologies of the BHJ thin films were characterized by transmission electron microscopy (TEM, JEM-ARM200F, Japan). The GIWAXS characterization of the thin films was performed at the Advanced Light Source (Lawrence Berkeley National Laboratory) on beamline 7.3.3, with the incidence angle of 0.16°, and the beam energy of 10 keV. Samples were prepared under optimal conditions on the Si/PEDOT:PSS substrates.

**Device Characterization.** The current-voltage (*J*-*V*) curves were measured with Keithley 2400 Source under the illumination of AM 1.5G irradiation (100 mW cm^-2^) using a 150 W solar simulator (DM-40S3, SAN-EI ELECTRIC, Japan) in glove box at room temperature. The light intensity was determined by a 2 × 2cm^2^ standardized monosilicon cell (Oriel PN 91150V, Newport, USA.) calibrated by the National Renewable Energy Laboratory (NREL). The external quantum efficiency (EQE) measurement was performed using an Enlitech EQE system (Enlitech QE-M110) with a Si diode as reference cell. The *J*–*V* curves were measured along the forward scan direction from -0.2 to 1.0 V or the reverse scan direction from 1.0 to -0.2 V, yielding identical results. The scan speed and dwell times were fixed at 0.015 V s^−1^ and 20 ms, respectively.

**Ultraviolet photoelectron spectroscopy measurement.** Ultraviolet Photoelectron Spectroscopy analysis was conducted using an AXIS Ultra DLD spectrometer (Kratos Analytical Inc., Mancheser, UK) with a He discharge UV lamp with He I radiation (incident photo energy, 21.22 eV), a hemispherical analyzer operating in the fixed analyzer transmission mode and the standard aperture (analysis area: 0.3 mm * 0.7 mm).

**EQE_EL_ measurement.** The EQE_EL_ was recorded with an in-house-built system comprising a standard silicon photodiode 1010B, Keithley 2400 source meter (for supplying voltages and recording injected currents), and Keithley 6482 picoammeter (for measuring the emitted light intensity).

**Highly sensitive EQE.** The halogen light source (LSH-75, Newport) passed through the monochromator(CS260-RG-3-MC-A, Newport) to form monochromatic light, which was focused on the device to generate electrical signals. Signals were finally collected by the front-end current amplifier (SR570, Stanford) and phase-locked amplifier (Newport). A corrected silicon solar cell (S1337-1010BR) was used as a standard detector.

**Impedance Spectroscopy.** Impedance measurements were carried out by illumination with a 1.5G illumination source (1000 W m-2) using a Solar Simulator. Impedance spectra were measured for different light intensities by applying a small voltage perturbation (10 mV rms) at frequencies from 8 MHz to 50 Hz, for different bias voltages. To measure in open circuit voltage conditions, a bias voltage equals to *V*_OC_ at each light intensity was applied. These measurements were performed with LCR-IM3536 equipped with a frequency analyzer module, always at room temperature. The data was collected for 10 average. Recombination resistance and chemical capacitance were directly extracted from the low-frequency region.

**Transient absorption spectroscopy.** For femtosecond transient absorption spectroscopy, the fundamental output from Yb:KGW laser (1030 nm, 220 fs Gaussian fit, 100 kHz, Light Conversion Ltd) was separated to two light beam. One was introduced to NOPA (ORPHEUS-N, Light Conversion Ltd) to produce a certain wavelength for pump beam (here we use 550 and 750 nm, 30 fs pulse duration), the other was focused onto a YAG plate to generate white light continuum as probe beam. The pump and probe overlapped on the sample at a small angle less than 10°. The transmitted probe light from sample was collected by a linear CCD array.

**Supplementary Tables**

**Table S1.** Photovoltaic parameters of PM6:Y6:ITC6-IC devices with different mixing ratio of acceptors under illumination of AM 1.5G, 100 mW/cm^2^. The average parameters were calculated from 40 devices, with the area of 0.032 cm^2^. Values outside the parentheses denote the best optimal results.

| **PM6:Y6:ITC6-IC** | ***V*_OC_**  **(V)** | ***J_SC_***  **(mA·cm^-2^)** | **FF**  **(%)** | **PCE**  **(%)** |
| --- | --- | --- | --- | --- |
| 1:1.2:0 | 0.843  (0.841±0.002) | 25.36  (25.24±0.16) | 78.32  (77.50±0.76) | 16.74  (16.45±0.29) |
| 1:1.1:0.1 | 0.854  （0.854±0.002） | 25.59  （25.40±0.33） | 78.82  （78.82±0.62） | 17.22  （17.09±0.20） |
| 1:1:0.2 | 0.866  (0.866±0.001) | 25.49  (24.45±0.15) | 79.05  (78.17±0.38) | 17.46  (17.23±0.23) |
| 1:0.9:0.3 | 0.866  （0.866±0.001） | 24.67  （24.54±0.28） | 76.58  （75.94±0.56） | 16.36  （16.14±0.26） |
| 1:0:1.2 | 1.026  （1.026±0.001） | 14.24  （14.11±0.40） | 58.86  （58.55±0.80） | 8.60  （8.47±0.18） |

Table S2. The *V*_OC_ values after mixing phase manipulation from some previous reports (the basic system is PM6:Y6)

| **No.** | **Third Component** | **Ratio** | ***V*_OC_ (V)** | **Ref** |
| --- | --- | --- | --- | --- |
| 1 | Y6-1O | 1:0.96:0.24 | 0.867 | [1] |
| 2 | PM7&PCBM | 0.8:0.2:1.2:0.25 | 0.859 | [2] |
| 3 | AQx-3 | 1:0.8:0.4 | 0.866 | [3] |
| 4 | IDYP-4F | 1:1.1:0.1 | 0.863 | [4] |
| 5 | BTTPC | 1:1.26:0.14 | 0.860 | [5] |
| 6 | DTTC-4Cl-C9 | 1:1.02:0.18 | 0.860 | [6] |
| 7 | ITIC-Th | 1:1.12:0.08 | 0.860 | [7] |

**Table S3.** The summary of the energy loss details for the binary and ternary devices.

| **PM6:Y6:A** | ***E_gap_* (eV)** | ***qV_oc_* (eV)** | ***E*_loss_ (eV)** | **Δ*E*_1_ (eV)** | **Δ*E*_2_ (eV)** | **Δ*E*_3_ (eV)** |
| --- | --- | --- | --- | --- | --- | --- |
| **None** | 1.414 | 0.841 | 0.573 | 0.264 | 0.047 | 0.262 |
| **IT-M** | 1.432 | 0.872 | 0.560 | 0.265 | 0.051 | 0.244 |
| **ITIC** | 1.430 | 0.867 | 0.563 | 0.265 | 0.05 | 0.248 |
| **ITC6-IC** | 1.422 | 0.866 | 0.556 | 0.265 | 0.047 | 0.244 |
| **IT-2Cl** | 1.422 | 0.858 | 0.564 | 0.265 | 0.042 | 0.257 |
| **IDIC** | 1.421 | 0.854 | 0.567 | 0.265 | 0.056 | 0.246 |
| **ITC6-4F** | 1.422 | 0.851 | 0.570 | 0.264 | 0.049 | 0.257 |
| **IT-4F** | 1.426 | 0.847 | 0.579 | 0.265 | 0.051 | 0.263 |
| **IT-4Cl** | 1.430 | 0.845 | 0.585 | 0.265 | 0.049 | 0.271 |

**Table S4.** Summary of the fitting results of IS data.

| **PM6:Y6:A** | ***E*_U_**  **[meV]** | ***N*_t_**  **[cm^-3^]** | **δ**  **[meV]** |
| --- | --- | --- | --- |
| **None** | 24.5 | 3.63*10^20^ | 58.0 |
| **IT-M** | 26.2 | 1.83*10^20^ | 58.8 |
| **ITIC** | 25.9 | 2.14*10^20^ | 58.6 |
| **ITC6-IC** | 25.4 | 2.38*10^20^ | 57.0 |
| **IT-2Cl** | 26.6 | 2.66*10^20^ | 57.1 |
| **IDIC** | 25.2 | 2.47*10^20^ | 56.5 |
| **ITC6-4F** | 26.5 | 2.79*10^20^ | 57.0 |
| **IT-4F** | 26.0 | 2.96*10^20^ | 58.2 |
| **IT-4Cl** | 26.3 | 3.19*10^20^ | 58.5 |

**Table S5.** Summary of hole transfer time and electron transfer time derived by fitting the corresponding TA kinetics (*τ*_1_ and *τ*_2_ are from the hole side, *τ* is from the electron side).

| **PM6:Y6:A** | **A_1_ (%)** | ***τ*_HT_ (ps)** | **A_2_ (%)** | ***τ*_HD_ (ps)** | ***τ*_ET_ (ps)** |
| --- | --- | --- | --- | --- | --- |
| **None** | 57.96 | 0.312±0.014 | 45.04 | 12.18±0.98 | 0.336±0.006 |
| **IT-M** | 55.00 | 0.393±0.026 | 45.00 | 16.73±1.00 | 0.376±0.009 |
| **ITIC** | 55.21 | 0.301±0.009 | 44.79 | 14.02±1.37 | 0.544±0.019 |
| **ITC6-IC** | 54.98 | 0.268±0.013 | 45.02 | 10.86±1.35 | 0.422±0.045 |
| **IT-2Cl** | 55.45 | 0.287±0.010 | 44.55 | 13.72±0.92 | 0.403±0.067 |
| **IDIC** | 55.00 | 0.320±0.011 | 45.00 | 12.99±1.09 | 0.395±0.009 |
| **ITC6-4F** | 55.02 | 0.339±0.008 | 44.98 | 13.96±1.73 | 0.339±0.007 |
| **IT-4F** | 55.00 | 0.348±0.008 | 45.00 | 14.83±1.38 | 0.337±0.009 |
| **IT-4Cl** | 55.04 | 0.407±0.012 | 44.96 | 14.58±1.31 | 0.337±0.008 |

**Table S6.** Summary of the fitting results of for BHJ devices.

| **PM6:Y6:A** | **Pi-pi Peak Height**  **(a.u.)** | **Pi-pi Peak Area** | **Y6 (11-1)**  **Peak Height (a.u.)** | **Y6 (11-1)**  **Peak Area** | **Y6 (11-1)**  **Peak CCL (A)** |
| --- | --- | --- | --- | --- | --- |
| **None** | 374.6 | 157.5 | 76.2 | 11.6 | 58.1 |
| **IT-M** | 215.6 | 92.4 | 44.4 | 9.8 | 40.4 |
| **ITIC** | 322.1 | 140.7 | 80.8 | 24.2 | 29.6 |
| **ITC6-IC** | 343.3 | 153.8 | 93.5 | 29.2 | 28.5 |
| **IT-2Cl** | 223.1 | 99.4 | 60.6 | 18.8 | 28.7 |
| **IDIC** | 229.3 | 96.5 | 49.4 | 8.0 | 55.1 |
| **ITC6-4F** | 287.7 | 122.6 | 71.6 | 15.8 | 40.3 |
| **IT-4F** | 230.3 | 103.3 | 53.8 | 11.6 | 41.3 |
| **IT-4Cl** | 233.7 | 103.7 | 58.1 | 16.0 | 32.3 |

**Table S7.** Summary of the fitting results of for acceptor-only devices.

| **Y6:A** | **Y6 (11-1) Peak g-factor** | **Y6 (11-1)**  **Peak CCL (A)** | **Y6 (11-1)**  **Peak Height (a.u.)** | **Y6 (11-1)**  **Peak Area** |
| --- | --- | --- | --- | --- |
| **None** | 0.11 | 157.8 | 355.7 | 20.0 |
| **IT-M** | 0.16 | 83.0 | 222.4 | 23.8 |
| **ITIC** | 0.17 | 69.6 | 179.0 | 22.8 |
| **ITC6-IC** | 0.30 | 55.9 | 122.0 | 19.4 |
| **IT-2Cl** | 0.21 | 49.2 | 156.3 | 28.2 |
| **IDIC** | 0.21 | 50.3 | 124.7 | 22.0 |
| **ITC6-4F** | 0.18 | 65.0 | 150.6 | 20.58 |
| **IT-4F** | 0.19 | 57.6 | 142.1 | 21.9 |
| **IT-4Cl** | 0.22 | 45.47 | 196.0 | 38.3 |

**Supplementary Figures**

**
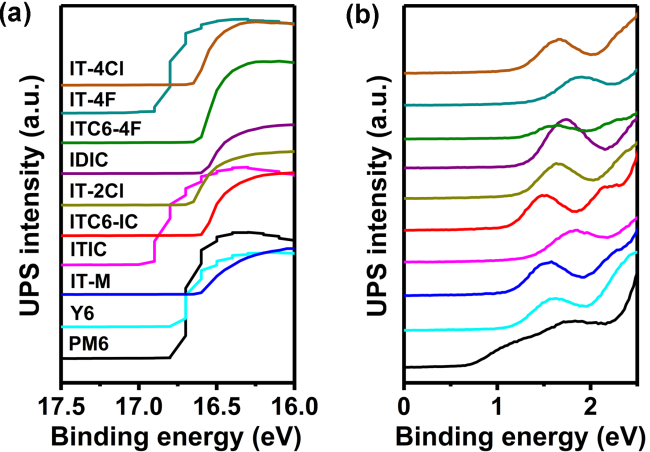
**

**Figure S1. UPS spectra of neat films.** UPS spectra corresponding to (a) the secondary electron cutoff and (b) valence band maximum of materials used in this study.





**Figure** S**2.** Current density-voltage (*J-V*) curves of PM6:Y6 based binary and ternary devices.





**Figure S3.** Photovoltaic parameters of PM6:Y6:ITC6-IC devices The evolution of average device parameters (PCE, *V*_OC_, *J*_SC_, and FF) as a function of ITC6-IC contents. The average values and standard deviations were derived from 40 devices.


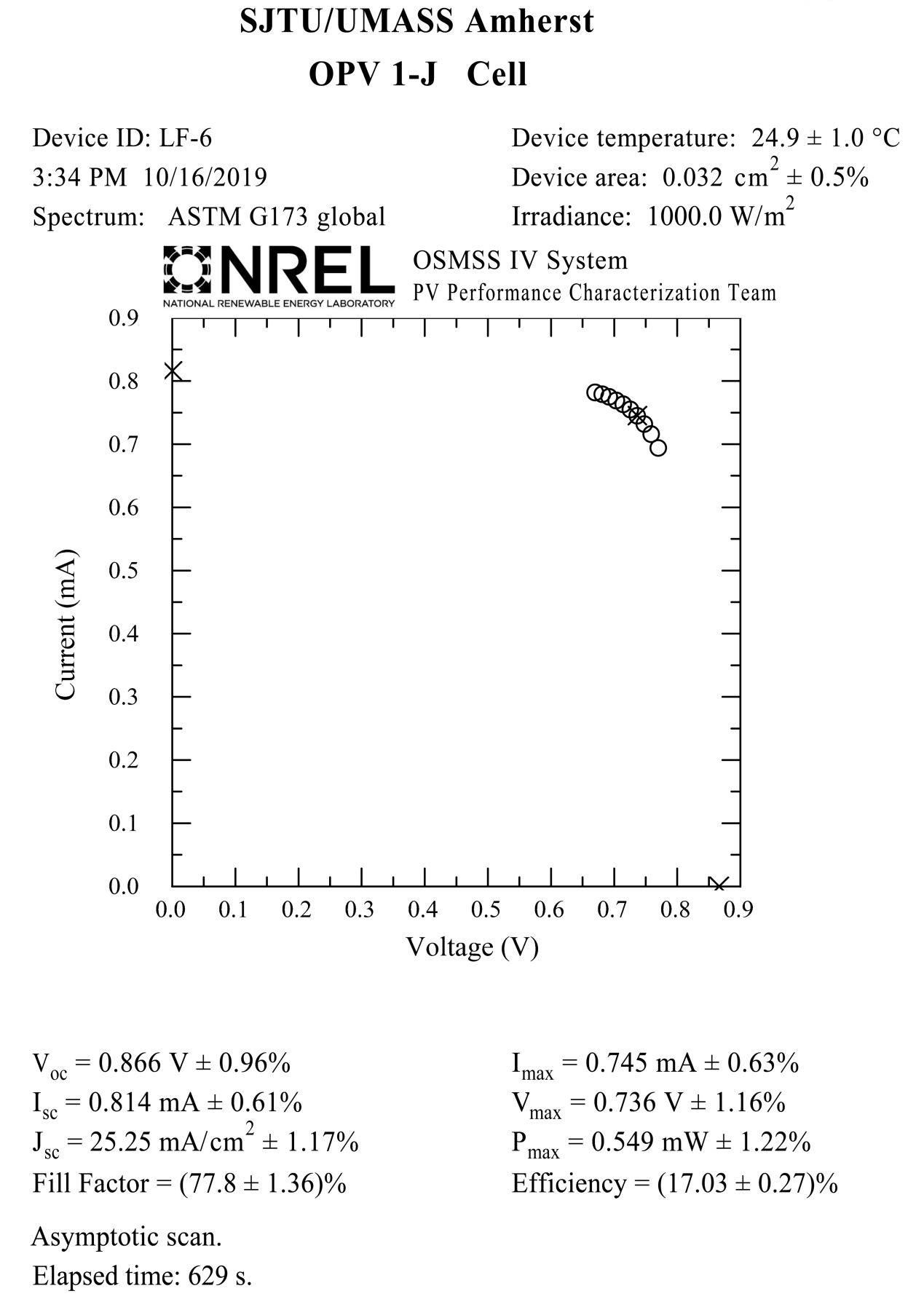


**Figure S4.** NREL certification report of PM6:Y6:ITC6-IC based device.





**Figure S5.** External quantum efficiency (EQE) spectra. EQE spectra of devices based on PM6:Y6:A based ternary devices (A denotes ITIC and its derivatives).





**Figure S6.** The miscibility parameters of PM6, Y6 and ITIC, where *δ*_h_ is the contribution of hydron bonding, and *δ*_v_ is the contribution of dispersion and polarity. The dash circles are the miscibility range, and the radii are 5 and 10 J^0.5^ cm^-1.5^ for polymer and NFA, respectively.


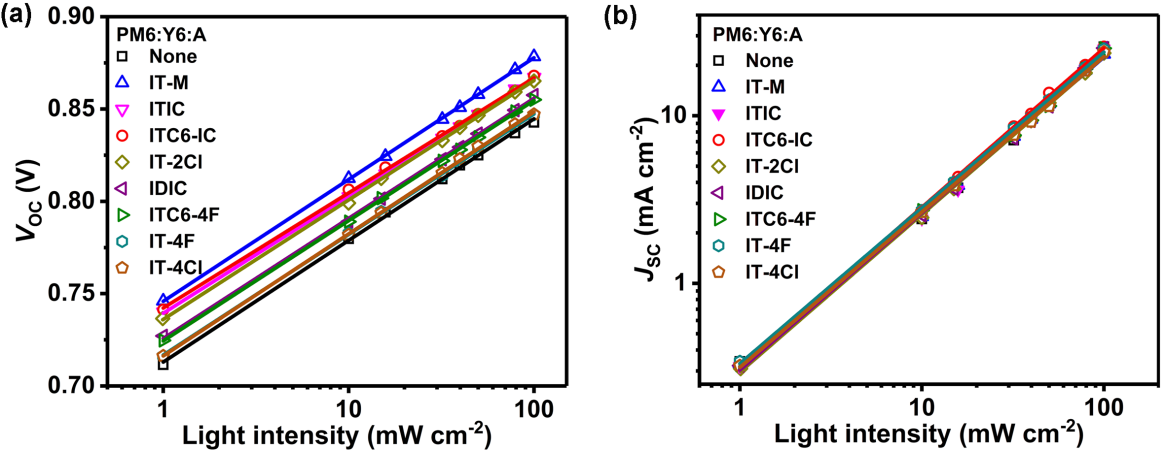


**Figure S7.** Measurement of light intensity-dependent *V*_OC_ (a) and *J*_SC_ (b) for PM6:Y6:A based devices.


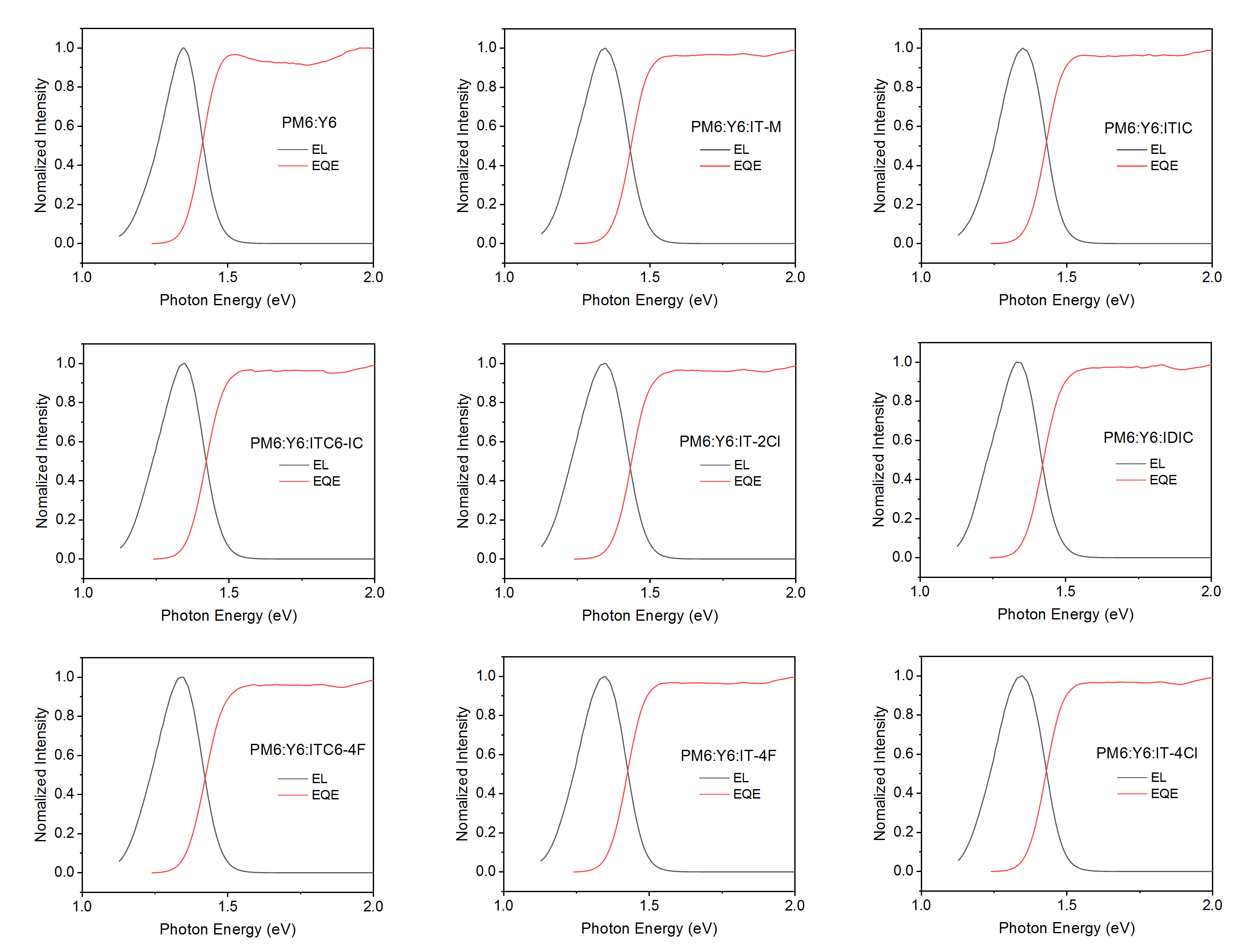


**Figure S8. Energy loss analysis.** The electroluminescence spectra (EL, black solid line), the external quantum efficiency (EQE_PV_, black solid line), and intersection to determine *E*_g_.





**Figure S9.** The relationship between *V*_OC_ and radiative energy loss.

**
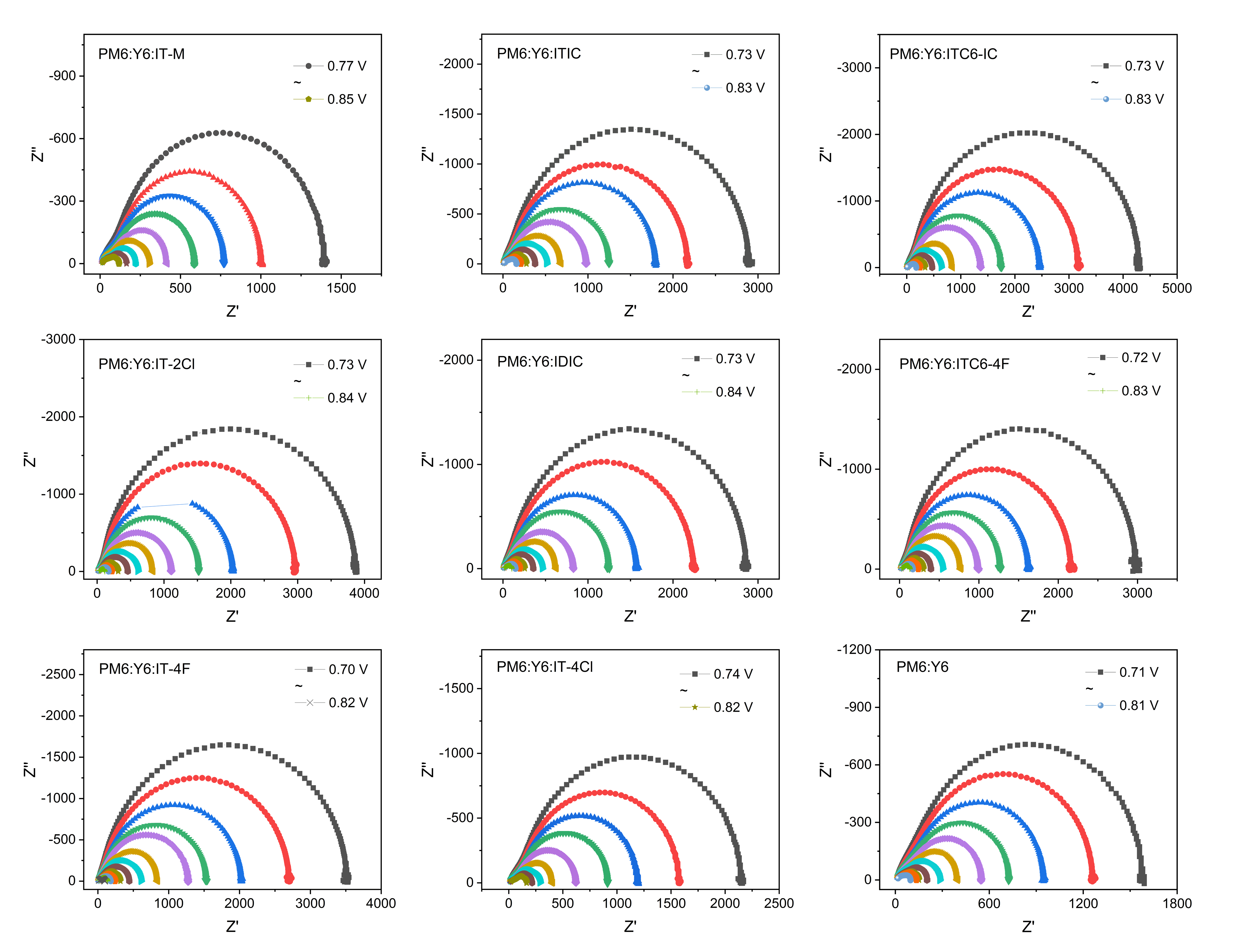
**

**Figure S10.** The impedance spectra of the devices with different irradiation. A reverse DC bias is applied to obtain the open-circuit condition.

**
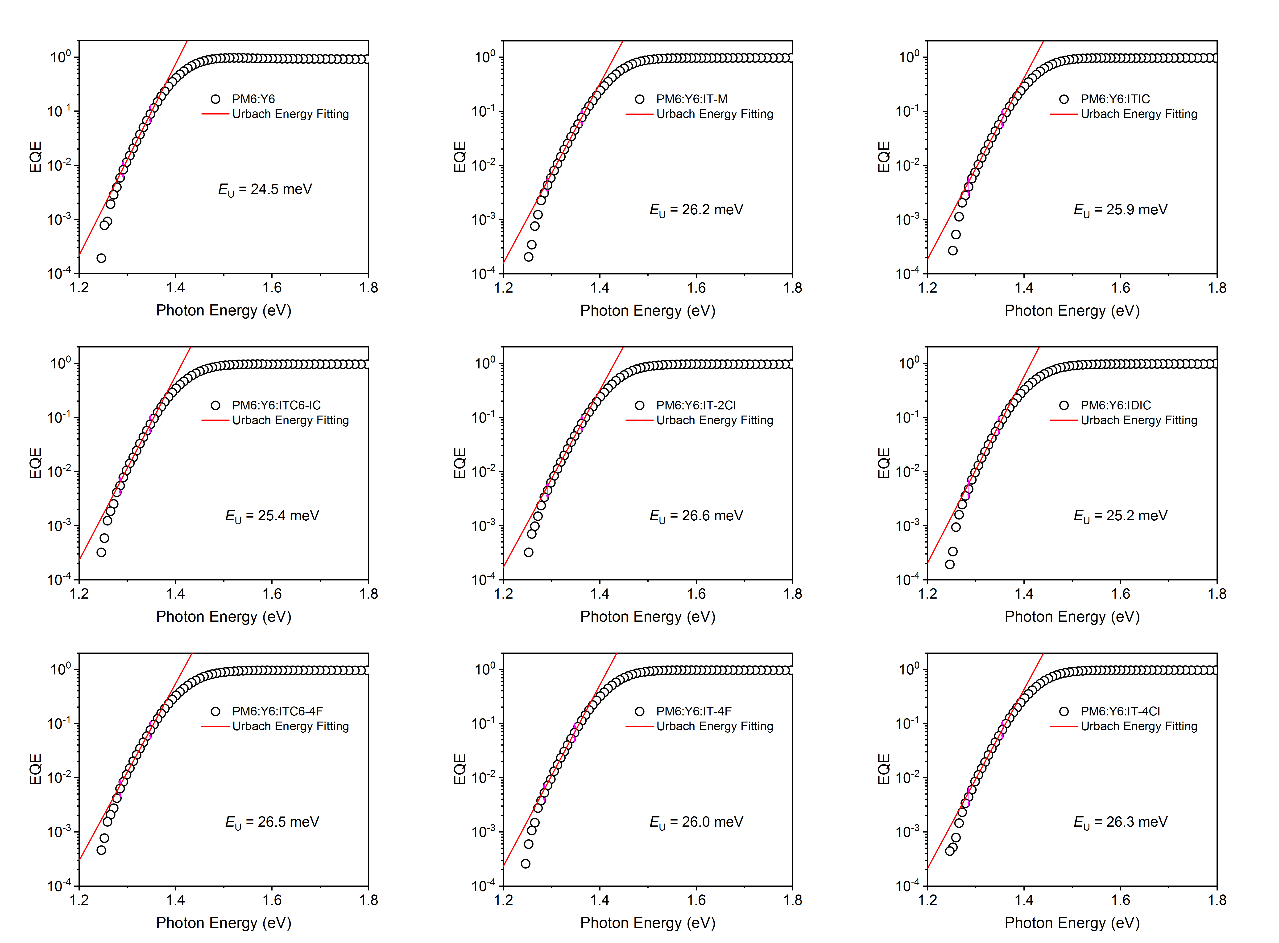
**

**Figure S11.** The Urbach Energy fitting of the energy onset of the s-EQE spectra.

**
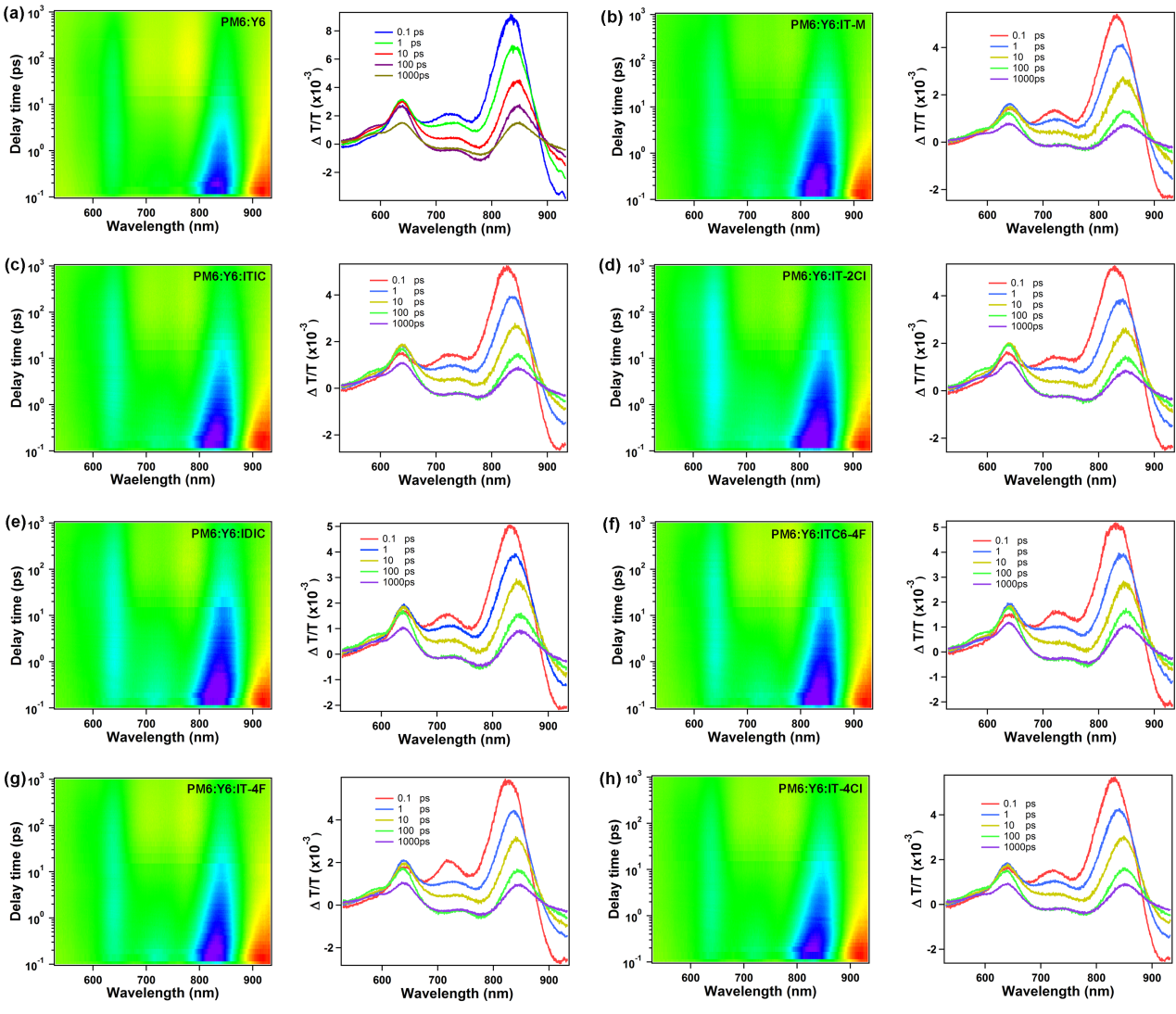
**

**Figure S12.** Transient absorption (TA) for hole transfer kinetics. 2D color images and corresponding TA spectra: (a) PM6:Y6, (b) PM6:Y6:IT-M, (c) PM6:Y6:ITIC, (d) PM6:Y6:IT-2Cl, (e) PM6:Y6:IDIC, (f) PM6:Y6:ITC6-4F, (g) PM6:Y6:IT-4F, and (h) PM6:Y6:IT-4F.


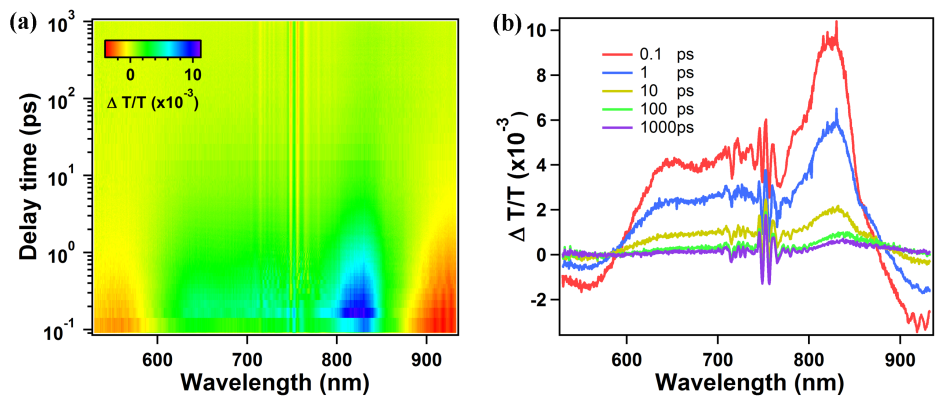


**Figure S13.** Transient absorption (TA) for Y6:ITC6-IC film. (a) 2D color images and (b) corresponding TA spectra of Y6:ITC6-IC films.


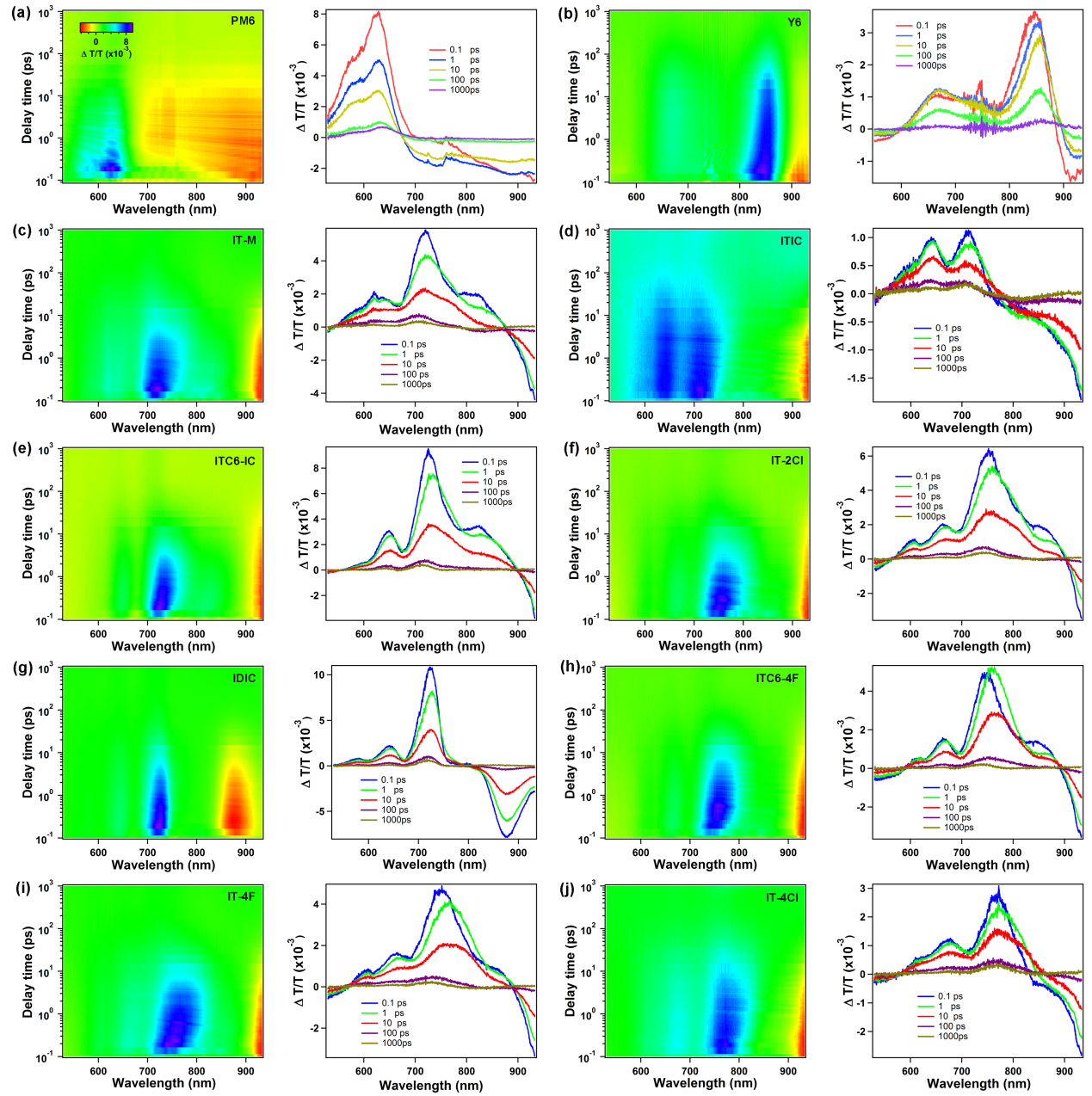


**Figure S14.** Transient absorption (TA) for neat films. 2D color images and corresponding TA spectra: (a) PM6, (b) Y6, (c) IT-M, (d) ITIC, (e) ITC6-IC, (f) IT-2Cl, (g) IDIC, (h) ITC6-4F, (i) IT-4F, and (j) IT-4Cl.


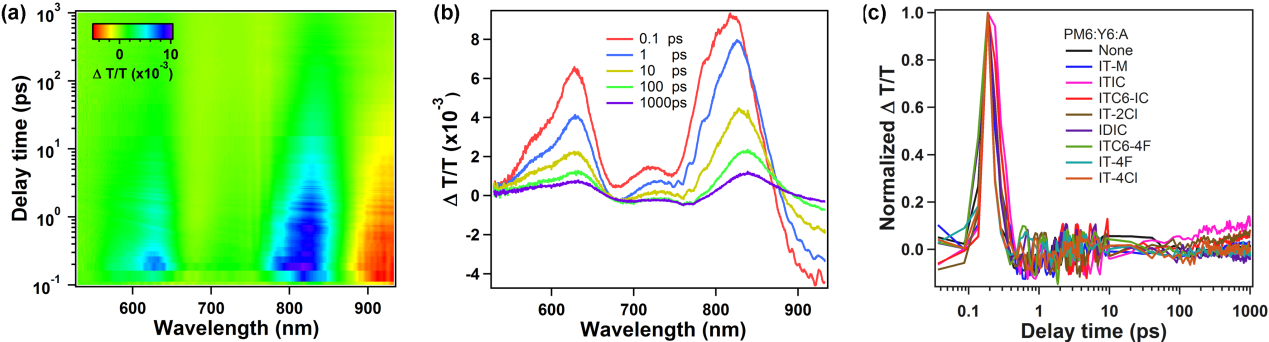


**Figure S15.** Transient absorption (TA) with excitation wavelength of 550 nm. (a) 2D TA color plot of PM6:Y6:ITC6-IC based ternary films. **(b)** Representative TA spectra at indicated delay times. **(c)** TA kinetics of PM6:Y6:A films used to extract electron transfer rate.


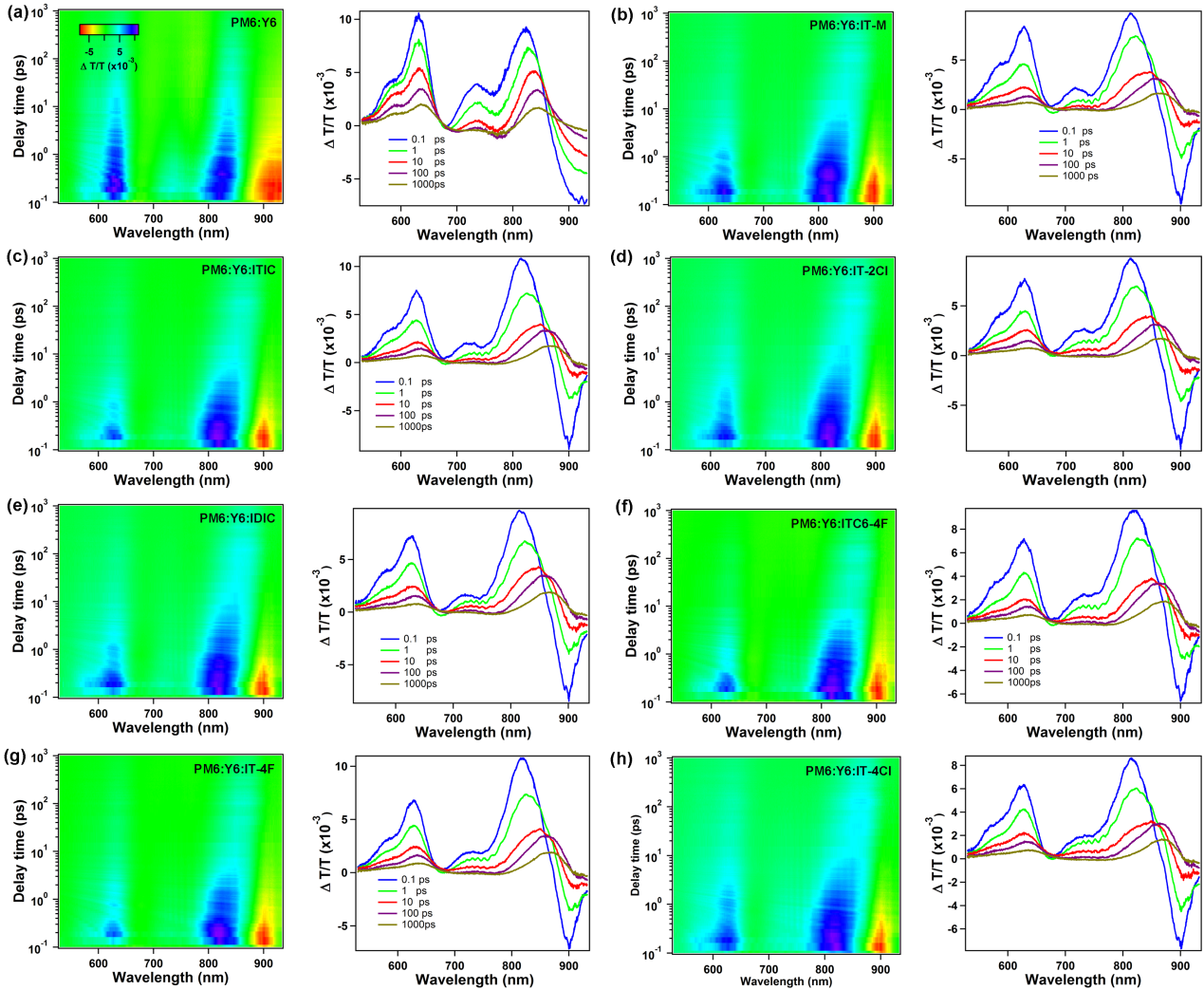


**Figure S16.** Transient absorption (TA) for electron transfer kinetics. 2D color images and corresponding TA spectra: (a) PM6:Y6, (b) PM6:Y6:IT-M, (c) PM6:Y6:ITIC, (d) PM6:Y6:IT-2Cl, (e) PM6:Y6:IDIC, (f) PM6:Y6:ITC6-4F, (g) PM6:Y6:IT-4F, and (h) PM6:Y6:IT-4F.


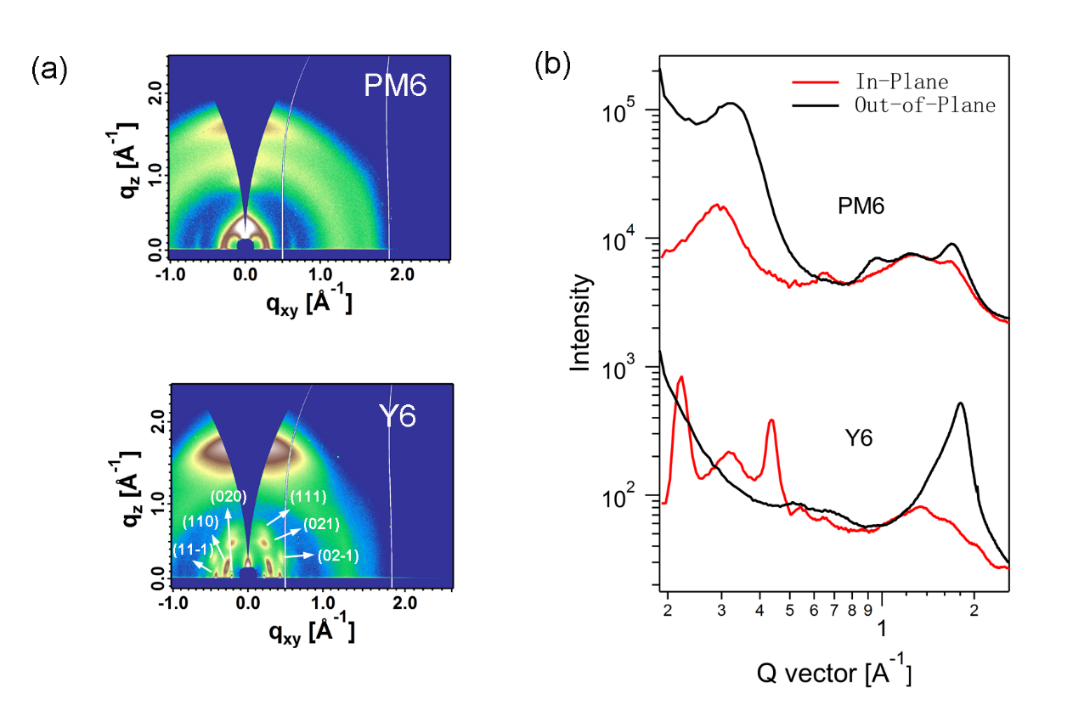


**Figure S17.** (a) 2D GIXD image of neat PM6 and Y6 films, and (b) the corresponding 1D profile. The black lines represent the out-of-plane direction, and the rede lines represent the in-plane direction. The miller index of Y6 crystal is attached.


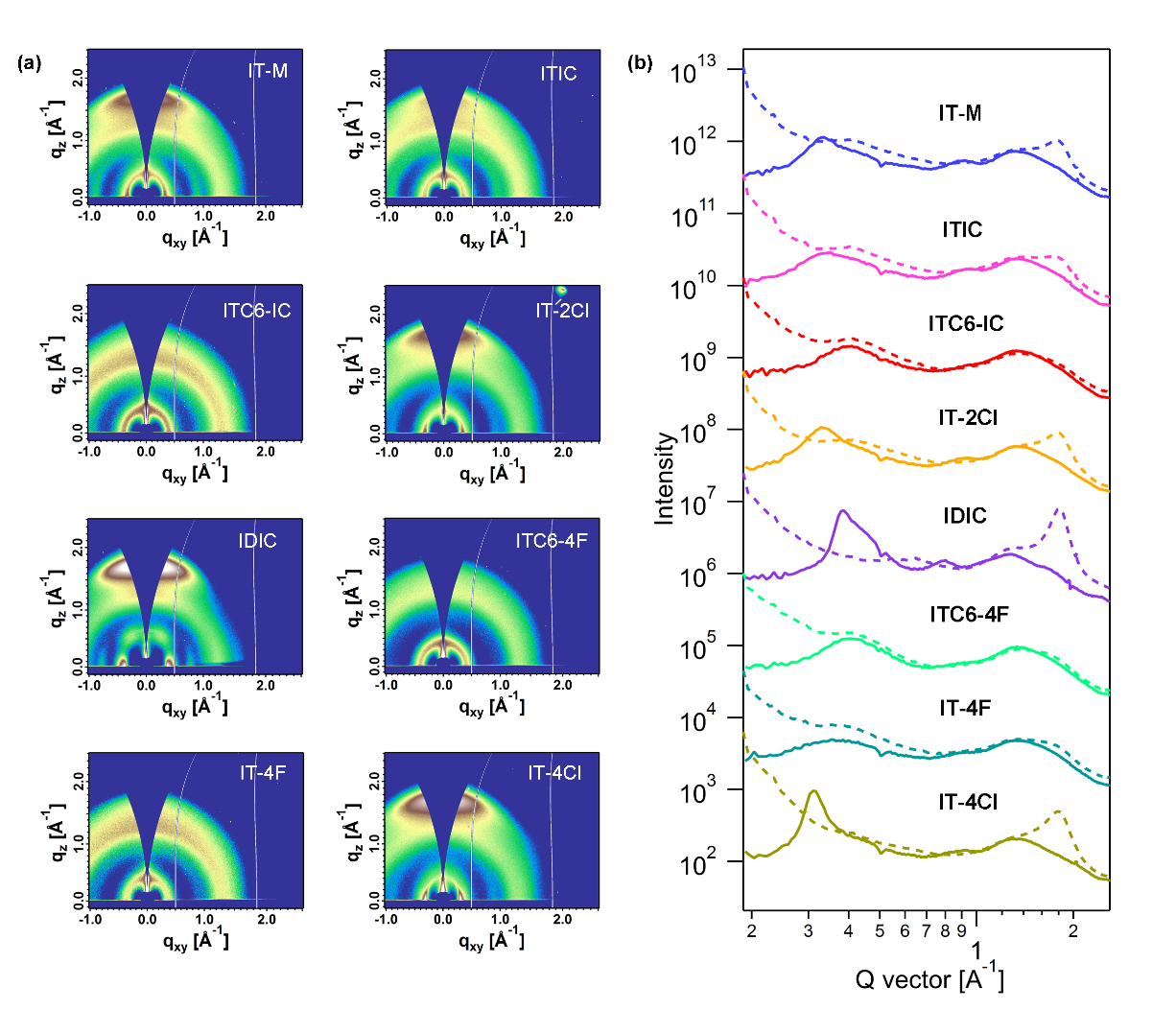


**Figure S18.** (a) 2D GIXD images of ITIC derivatives meat films, and (b) its corresponding 1D profiles. The dashed lines represent the out-of-plane direction, and the solid lines represent the in-plane direction.


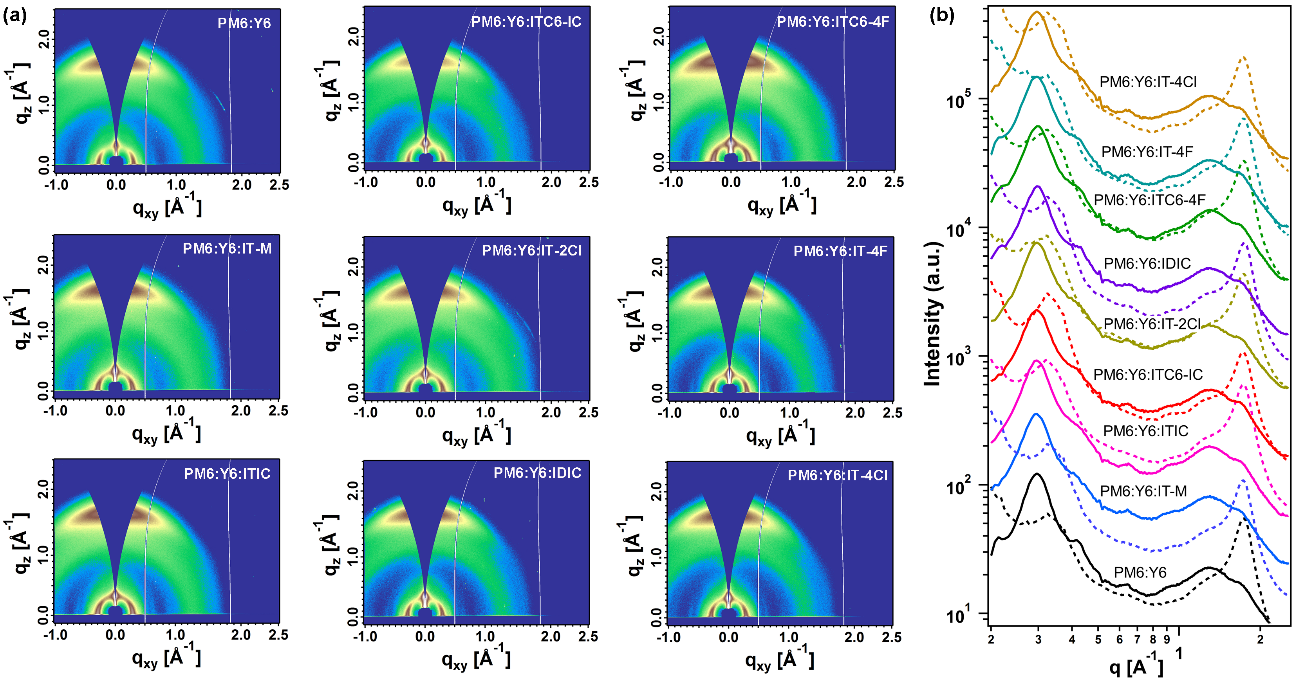


**Figure S19.** (a) 2D GIXD images of PM6:Y6 binary and PM6:Y6:A ternary blend films, and (b) its corresponding 1D profiles. The dashed lines represent the out-of-plane direction, and the solid lines represent the in-plane direction.


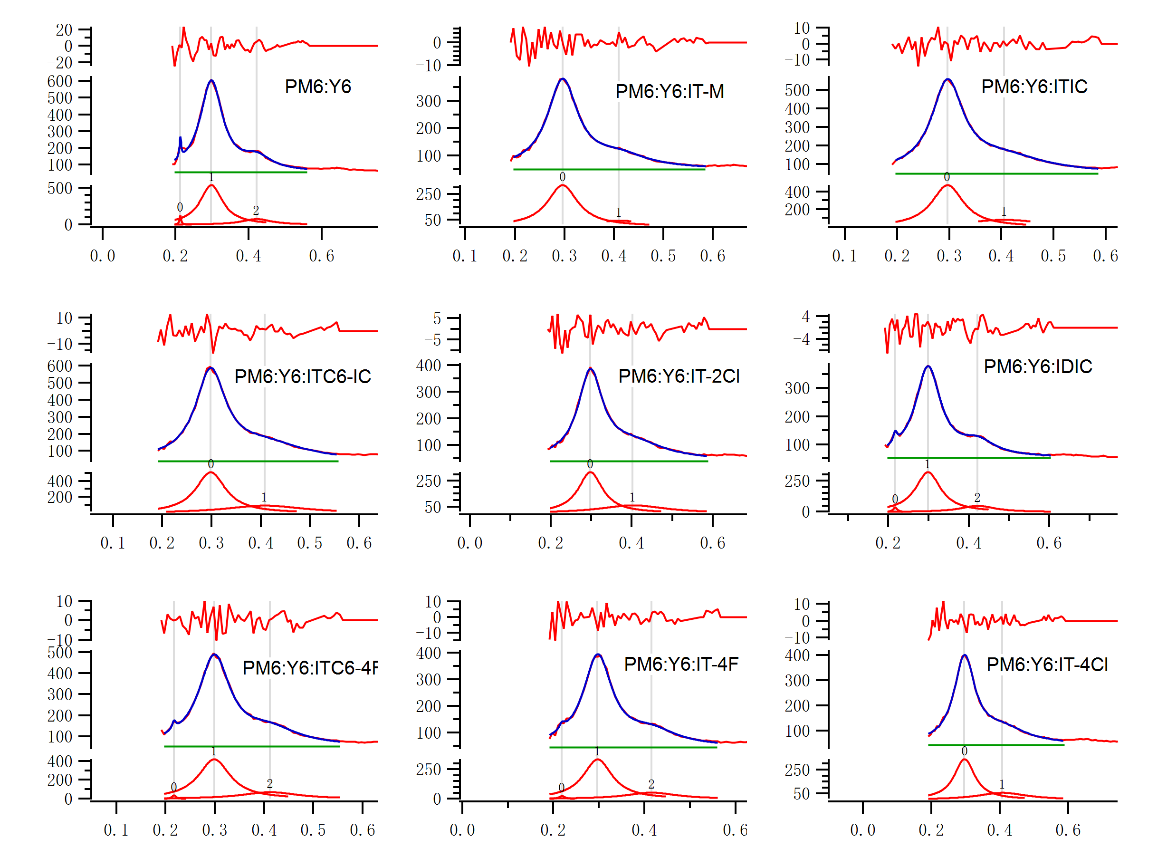


**Figure S20.** The multiple Lorentzian fitting of the line cuts along the IP direction for the BHJ thin films.


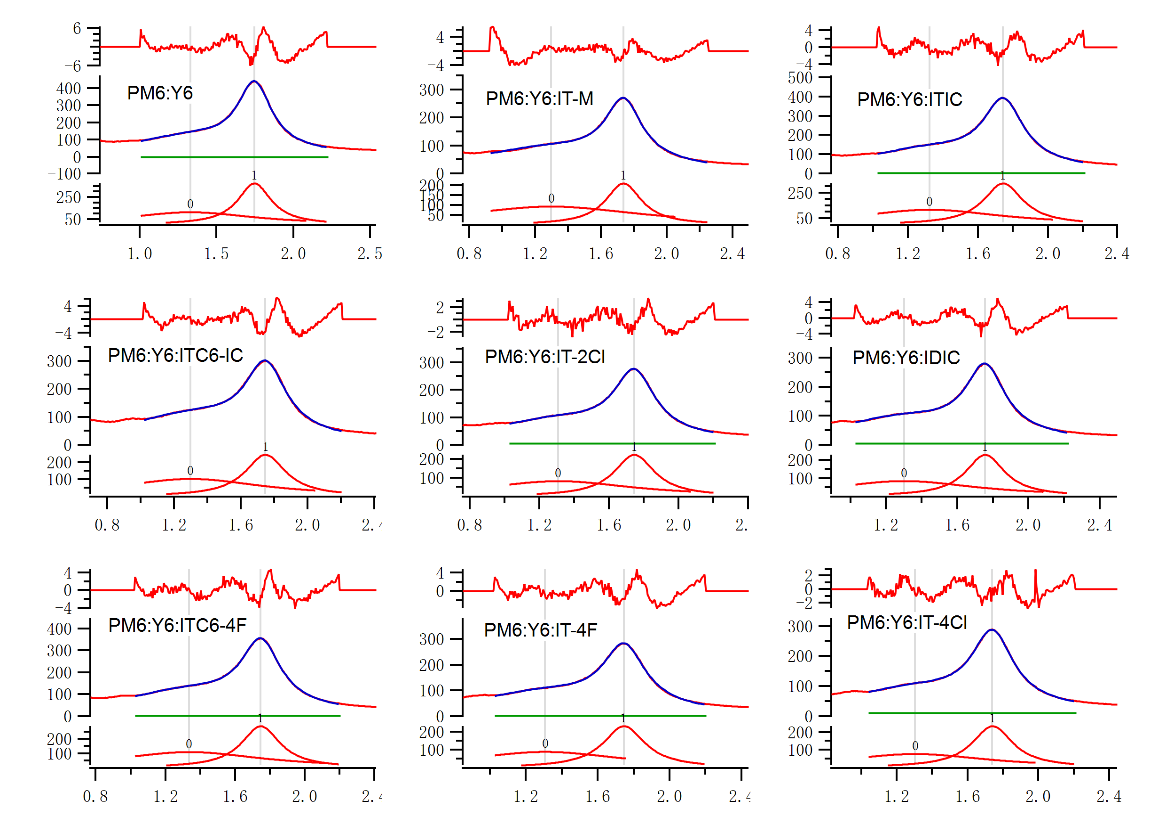


**Figure S21.** The multiple Lorentzian fitting of the line cuts along the OOP direction for the BHJ thin films.





**Figure S22.** The 2D relevance between exciton diffusion time and Y6 (11-1) peak CCL and Area in IP line cuts of BHJ devices. The color bar is used to reflect the value of exciton diffusion time in the 2D map, and the change from blue to red represents the improvement.


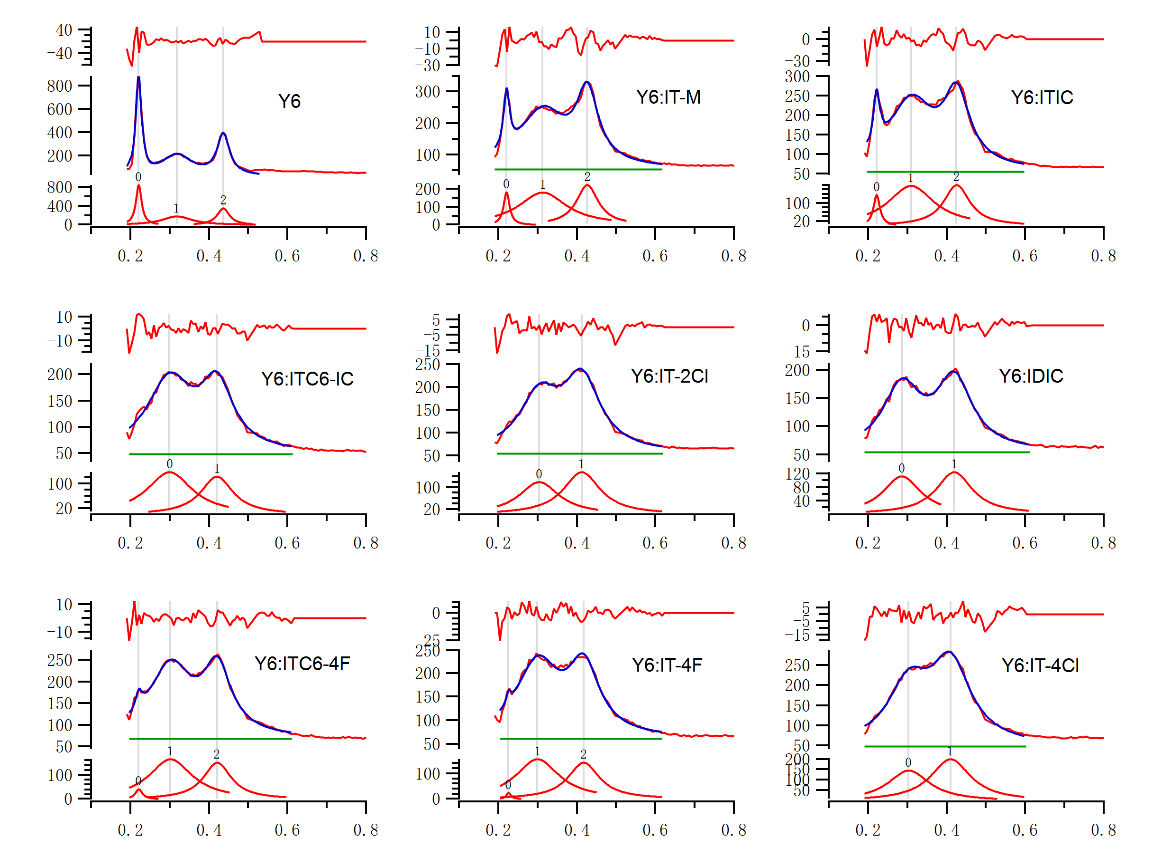


**Figure S23.** The multiple Lorentzian fitting of the line cuts along the IP direction for the acceptor-only thin films.


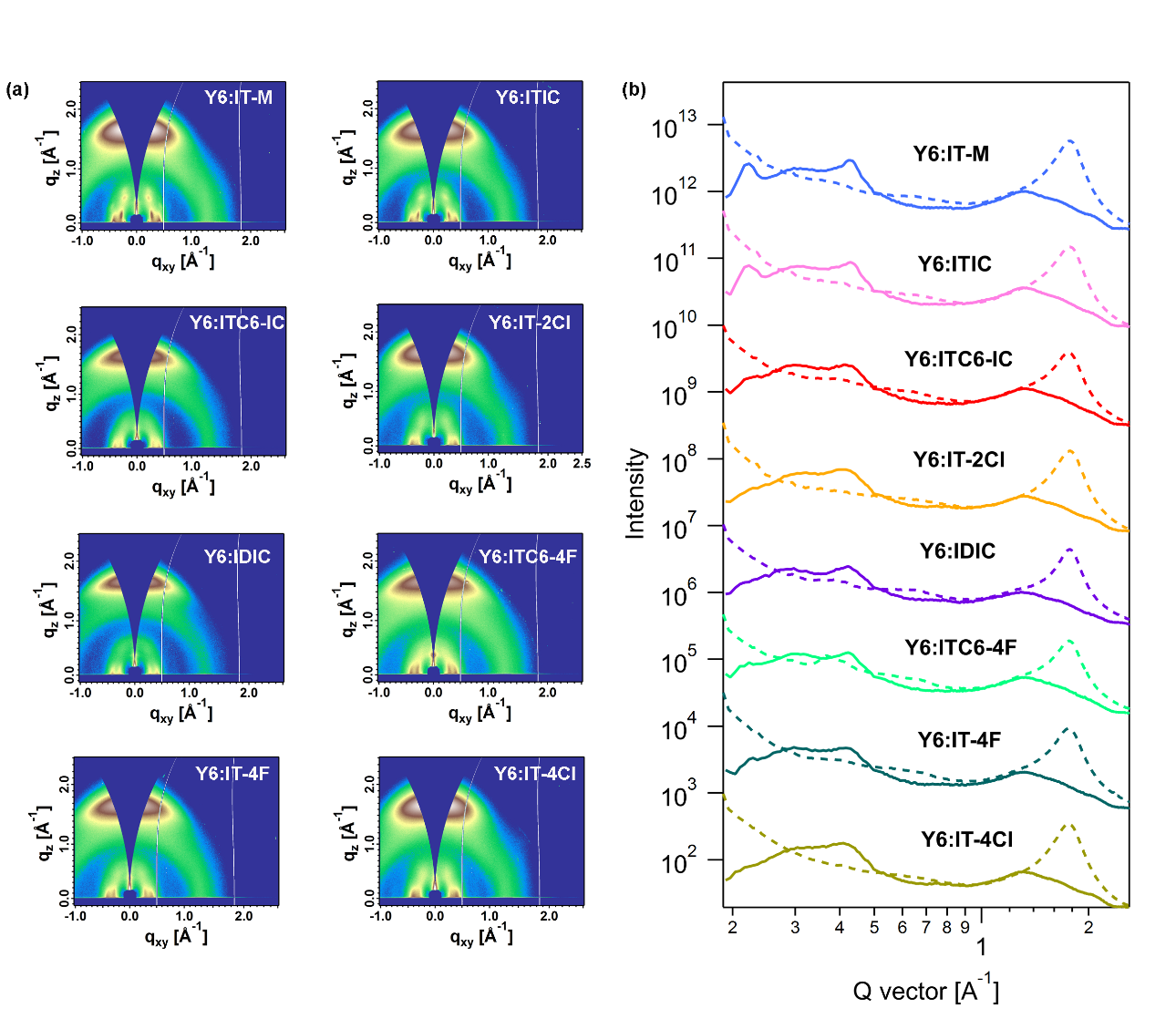


**Figure S24.** (a) 2D GIXD images of Y6:A thin films, and (b) its corresponding 1D profiles. The dashed lines represent the out-of-plane direction, and the solid lines represent the in-plane direction.


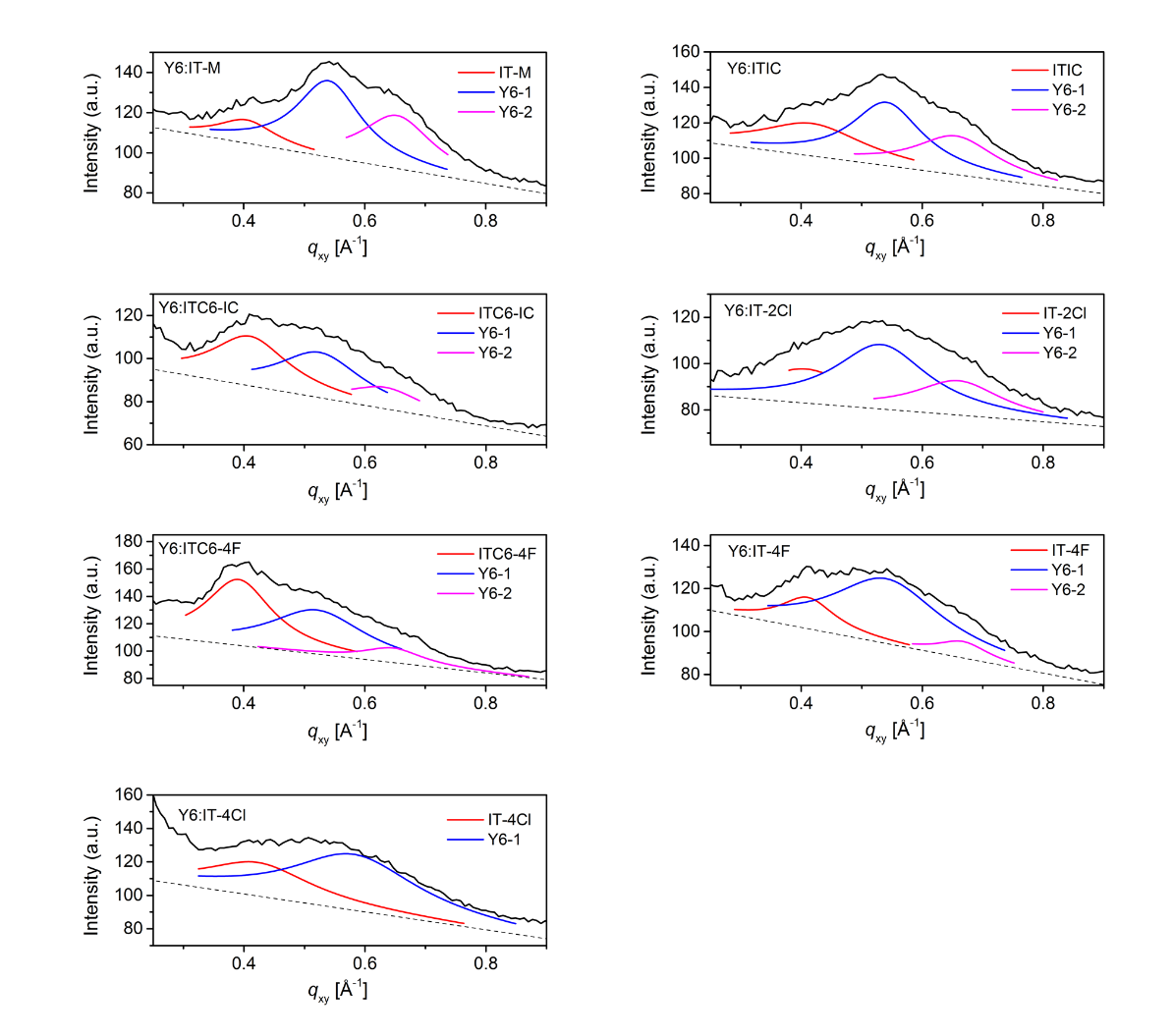


**Figure S25.** The low q region of the 75^o^ line cut profiles with Lorentzian fitting results (red line for the third component, blue line for Y6 (021) peak, purple line for Y6 (111) peak. It is difficult to accurately fit the Y6:IDIC curve in this region due to the signal overlap.





**Figure S26.** The relationship between the intensity of Y6 (021) peak in the 75^o^ direction and *J*_SC_, FF.

Reference

[1] K. B. Yu, W. Song, Y. F. Li et al., “Achieving 18.14% efficiency of ternary organic solar cells with alloyed nonfullerene acceptor. *Small Structure*, vol. 2, pp. 2100099-2100107, 2021.

[2] M. Zhang, L. Zhu, G. Q. Zhou et al., “Single-layered organic photovoltaics with double cascading charge transport pathways: 18% efficiencies,” *Nature Communications*, vol. 12, no. 1, pp. 309-319, 2021.

[3] F. Liu, L. Zhou, W. R. Liu et al., “Organic solar cells with 18% efficiency enabled by an alloy acceptor: a two‐in‐one strategy,” *Advanced Materials*, vol. 33, no. 27, pp. 2100830-2100837, 2021.

[4] J. Hu, Q. Guo, J. Fang et al., “High-performance alloy-like ternary organic solar cells with two compatible non-fullerene acceptors,” *Organic Electronics*, vol. 95, pp. 106201-106207, 2021.

[5] D. Wang, R. Qin, G. Q. Zhou et al., “High‐performance semitransparent organic solar cells with excellent infrared reflection and see-through functions,” *Advanced Materials*, vol. 32, no. 32, pp. 2001621-2001628, 2020.

[6] V. K. Karapala, T. -W. Chen, K. -J. Ma et al., “Exploring ternary organic solar cells for the improved efficiency of 16.5% with the compatible nonacyclic carbazole-based nonfullerene acceptors as the third component,” *ACS Applied Energy Materials*, vol. 4, no. 3, pp. 2847-2855, 2021.

[7] C. Q. Yan, R. J. Ma, G. L. Cai et al., “Reducing V_OC_ loss via structure compatible and high lowest unoccupied molecular orbital nonfullerene acceptors for over 17%‐efficiency ternary organic photovoltaics,” *Ecomat*, vol. 2, no. 4, pp. 12061-12071, 2020.
